# Radiolysis-Associated Decrease in Radiochemical Purity of ^177^Lu-Radiopharmaceuticals and Comparison of the Effectiveness of Selected Quenchers against This Process

**DOI:** 10.3390/molecules28041884

**Published:** 2023-02-16

**Authors:** Anton Larenkov, Iurii Mitrofanov, Ekaterina Pavlenko, Marat Rakhimov

**Affiliations:** State Research Center—Burnasyan Federal Medical Biophysical Center of Federal Medical Biological Agency, Zhivopisnaya Str., bld. 46, 123098 Moscow, Russia

**Keywords:** therapeutic radiopharmaceuticals, radiolysis, stability, radical scavengers, antioxidants, radiochemical purity, lutetium-177, PSMA-617, dosage form, absorbed dose

## Abstract

The radiolytic degradation of vector molecules is a major factor affecting the shelf life of therapeutic radiopharmaceuticals. The development of time-stable dosage forms of radiopharmaceuticals is the key to their successful implementation in clinical practice. Using [^177^Lu]Lu-PSMA-617 molecule as an example, the time dependence of the change in radiochemical purity (RCP, %) under radiolysis conditions was studied. The dependence of [^177^Lu]Lu-PSMA-617 radiolysis on parameters such as time, radionuclide activity, buffer agent concentration, precursor amount, and preparation volume was evaluated. It was shown that the absorbed dose was the dominant factor influencing the RCP. The RCP value is inversely proportional to the absorbed dose in the [^177^Lu]Lu-PSMA-617 preparation and has an exponential dependence. The lutetium-177 dose factor ψ (Gy·mL·MBq^−1^) and PSMA-617 concentration-dependent dose constant κ (Gy^−1^) were evaluated for absorbed dose estimation via computer modeling, chemical dosimetry, and radiochemical purity monitoring under various conditions. The further refinement and application of the dependencies found can be useful for predicting the RCP value at the stage of optimizing the composition of the finished dosage form of therapeutic radiopharmaceuticals. The influence of the buffer agent (sodium acetate) concentration on [^177^Lu]Lu-PSMA-617 radiolytic degradation was shown and should be considered both when developing a dosage form, and when comparing the results of independent studies. The effectiveness of the addition of various stabilizing agents, such as DMSA, cysteine, gentisic acid, vanillin, methionine, adenine, dobesilic acid, thymine, uracil, nicotinamide, meglumine, and mannitol, in suppressing the effects of radiolysis was evaluated.

## 1. Introduction

The use of radiopharmaceuticals for targeted radionuclide therapy (RNT), the effectiveness of which has been established during clinical trials, is currently recognized as a safe, economically, and logistically competitive method for the treatment of primary cancer, as well as distant metastases [1,2,3]. Currently, the segment of therapeutic radiopharmaceuticals comprises approximately one-third of the total number of radiopharmaceuticals in the global pharmacy market. However, this market segment is expected to grow at ˃5% compound annual growth rate in the coming years due to the increasing use of different therapeutic radionuclides in various pathological conditions [4,5,6]. Most radiopharmaceuticals used and developed today for RNT contain β^−^-emitting radionuclides, among which lutetium-177 seems to be one of the most promising, due to its nuclear properties, as well as the proven methods of obtaining it in required quantities and the results of its clinical use [1,7,8,9,10]. Two radiopharmaceuticals based on ^177^Lu have been approved for use in clinical practice with marketing authorization: [^177^Lu]Lu-DOTA-TATE (oxodotreotide, Lutathera^®^ [11]; RNT of neuroendocrine tumors) and [^177^Lu]Lu-PSMA-617 (vipivotide tetraxetan, Pluvicto™ [12]; RNT of metastatic castrate-resistant prostate cancer). Research and development of new therapeutic radiopharmaceuticals with ^177^Lu is actively ongoing.

Since for the preparation of monodosed therapeutic radiopharmaceuticals with lutetium-177 (such as [^177^Lu]Lu-PSMA-617 or [^177^Lu]Lu-DOTA-TATE), ^177^Lu activities from 2 to 8 GBq are used (usually 6–7.4 GBq [13,14,15]), and in some cases even higher (in [16], for example, a method for the synthesizing of a multidose preparation with an activity of ^177^Lu 40.0 ± 5.5 GBq is described), then the processes of radiolysis in the course of the synthesis and storage of these radiopharmaceuticals acquire a significant influence [17]. The radiolytic degradation of a vector molecule in radiopharmaceuticals under the action of radiation from an incorporated radionuclide can proceed in two ways: direct damage to the molecule by particles emitted by the radionuclide (lowest probability), and degradation of the molecule due to interaction with radicals formed during the radiolysis of water (highest probability). The radiolysis of water due to ionizing radiation results in the production of different chemically active species, such as solvated electrons, H^•^ atoms, ^•^OH radicals, H_3_O^+^ ions, and molecules (dihydrogen H_2_ and hydrogen peroxide H_2_O_2_), and can be written as:

(1) $$H_{2}O\;\overset{ionizing\;radiation}{\to }{\;e}_{\mathrm{aq}.}^{-},{\mathrm{OH}}^{•},H^{•},{\;H}_{3}O_{\mathrm{aq}.}^{+},{\;H}_{2},{\;H}_{2}O_{2}$$

The interaction of water radiolysis products with a vector molecule leads to structural transfigurations that dramatically change the initial pharmacokinetic and receptor-specific properties. To prevent the negative effects caused by various free radicals in different preparations, substances that are radical scavengers (antioxidants, which are often referred to as quenchers) can be used for stabilization.

One of the commonly used excipients to suppress radiolysis in radiopharmaceuticals is ethanol [18], which is well known as a radical scavenger [19,20,21]. For example, it was clearly shown that the RCP of the ^18^F-FDG preparation with the activity concentration of 14.8 GBq/mL in the absence of ethanol decreases to 86% or more within 10 h, and in the presence of 0.3 vol. % ethanol—just to 94% in total [22]. The effectiveness of ethanol to suppress radiolysis in radiopharmaceuticals has been demonstrated for various radiopharmaceuticals for both diagnostic and therapeutic purposes [23,24,25,26]. However, in the case of therapeutic radiopharmaceuticals, ethanol in the formulation (10% *v*/*v*) is present after the solid-phase purification and is used in combination with other antioxidants (e.g., ascorbic and gentisic acids).

Ascorbic acid has been proposed to be particularly useful as a buffer agent and radiolytic stabilizer simultaneously for the synthesis of metal-based radiopharmaceuticals [27,28]. The effectiveness of ascorbic acid as a radical scavenger has been demonstrated in the synthesis of ^177^Lu-radiopharmaceuticals used in clinical practice [29,30,31]. At the same time, the use of ascorbic acid in the composition of radiopharmaceuticals is associated with a number of difficulties, including stability, oxidation, and color change during synthesis (with heating) and during preparation storage [32,33,34], problems with quantitative analysis of the components of the reaction mixture and the final dosage form [35,36], and a decrease in radiochemical conversion during synthesis (in comparison with other buffer agents such as MES and ammonium acetate [37]). It has also been shown that the addition of ascorbic acid alone is not sufficient to maintain the radiochemical purity of [^177^Lu]Lu-PSMA-617 at the required level: using an activity of 740 MBq (1 mL, 10 μg of PSMA-617, 0.52 M ascorbate) at room temperature, the RCP decreased from approximately 98% to 80.4% ± 6.3% after 24 h [38]. However, when the radiopharmaceutical was frozen (−20 °C), the RCP was higher than 95% after 48 h. Hence, only the combination of ascorbic buffer, proper dilution (radioactive concentration ≤ 3.7 GBq/mL) and freezing resulted in the appropriate stability of [^177^Lu]Lu-PSMA-617 over a 48 h period [38].

Another extremely popular antioxidant used in radiopharmaceuticals is gentisic acid (2,5-dihydroxybenzoic acid, DHB). The effectiveness of gentisic acid in preventing radiolytic degradation in the final dosage form has been demonstrated for different ^177^Lu-radiopharmaceuticals used in clinical practice, such as [^177^Lu]Lu-PSMA-617, [^177^Lu]Lu-PSMA-I&T, and [^177^Lu]Lu-DOTATATE [16,39,40,41,42,43]. Furthermore, it has been shown that gentisic acid may improve the stability of radiopharmaceuticals even with α-emitting radionuclides: the addition of 0.1 M gentisic acid increases the stability of [^225^Ac]Ac-macropa-PSMA by preventing radiolytic degradation [44]. At the same time, the presence of 0.1 M ascorbic acid did not stabilize the ^225^Ac-labeled conjugates, leading to lower complex stability and transchelation. Ghosh et al. [45], using the compound [^177^Lu]Lu-RM1 (antagonist to gastrin-releasing peptide receptor) as an example, showed that samples (0.1 M acetate buffer, 0.74 GBq/mL activity concentration, 10.175–12.765 GBq/mmol molar activity) with ascorbic acid (500 μg) have a half-life of degradation around 39.2 h. Under the same conditions, the average half-life of the degradation of [^177^Lu]Lu-RM1 in the presence of 500 μg gentisic acid was 142.5 h. However, Trindade et al. used a DOTA-conjugated minigastrin analog—[^177^Lu]Lu-DOTA-H2MG11—and reported an undesired oxidative effect of gentisic acid [46]. The reduction of this effect, together with the suppression of radiolysis, was achieved using a mixture of gentisic acid (110 mM) and methionine (78 mM) as stabilizers. Mixtures of different antioxidants are often used in the formulation of radiopharmaceuticals, with the most popular being a combination of ascorbic and gentisic acids. The radiochemical purity of the [^177^Lu]Lu-PSMA-I&T (18–30 GBq of [^177^Lu]LuCl_3_, 500–800 μg of PSMA-I&T, 16.8 mg of gentisic acid, 371.2–531.2 mg of ascorbic acid, 32.4 mg of sodium acetate; volume of final dosage form—17–25 mL) 30 h after synthesis was ≥97% when stored at room temperature [47]. The effectiveness of gentisic and ascorbic acids mixtures in preventing radiolysis is explained by the fact that ascorbic acid reacts with the primary radicals of gentisic acid (^•^C_6_H_3_(OH)_3_COOH, C_6_H_3_(OH)(O^•^)COOH), reducing them and returning gentisic acid again into the reaction mixture and dosage form [48]. Additionally, it is a mixture of gentisic and ascorbic acids that is used in FDA/EMA-approved ^177^Lu-radiopharmaceuticals—Lutathera^®^ (370 MBq/mL of ^177^Lu, 0.63 mg/mL gentisic acid, 2.8 mg/mL ascorbic acid [49]) and Pluvicto™ (1000 MBq/mL of ^177^Lu, 0.39 mg/mL gentisic acid, 50.0 mg/mL sodium ascorbate [50])—with the stated shelf-life of 78 h and 120 h, respectively.

Chen et al. reported the results of the effect of various radical scavengers on maintaining the radiochemical purity of the bombesin derivative [^177^Lu]Lu-AMBA (0.15 mL, 555 MBq/mL of ^177^Lu, 6.6 mg/mL of stabilizer) [51]. After 24 h of storage, the studied substances were in the next row in terms of their effectiveness (RCP, %): ascorbic acid (83.6 ± 1.1%) > gentisic acid (72.1 ± 4.6%) = cysteine (71.6 ± 1.7%) > methionine (55.2 ± 2.5%) = ethanol (52.5 ± 2.8%); control—9.1 ± 2.3%. After 48 h of storage, the results were as follows: ascorbic acid (75.0 ± 1.3%) > cysteine (54.3 ± 0.9%) > gentisic acid (40.2 ± 6.5%) > methionine (33.5 ± 3.2%) > ethanol (21.6 ± 1.7%). According to the authors, Se-methionine turned out to be the most effective of the studied radioprotectors: for 0.5 mL preparations (814 MBq/mL ^177^Lu, 24 µg/mL AMBA, 0.04 M AcONa (pH 4.8), 0.2 mg/mL of radioprotector, and 0.8 mg/mL EDTA), the radiochemical purity after 24 h was 72.0 ± 2.6%, 26.6 ± 3.9%, and 19.5 ± 2.7% for Se-methionine, cysteine, and methionine, respectively. In the absence of a radioprotector, the RCP of the preparation after 24 h was 6.5 ± 7.9%, and when Se-methionine was used in combination with ascorbic acid, the RCP remained at 99.7 ± 0.2%.

To date, the main studies aimed at understanding and systematizing the processes of radiolysis in radiopharmaceuticals, as well as evaluating the effectiveness of various radioprotectors, include the work of the scientific team of Eric de Blois [17,52,53,54,55]. Thus, in [17], a study was conducted on the effectiveness of different radioprotectors and their mixtures as well as the effect of the final volume of the [^177^Lu]Lu-PSMA-617 preparation on the intensity of its radiolytic degradation. When labeling PSMA-617 under ‘preclinical’ conditions (i.e., 80 MBq/1.9 nmol in 0.14 mL), 10 mM methionine and 10% ethanol offered the best protection against radiolysis of all tested quenchers and quencher combinations. The RCP of the [^177^Lu]Lu-PSMA-617 preparation with a combination of gentisic acid and ascorbic acid (3.5 mM final concentration for both) was only 73.8 ± 3.1% after 24 h. The high RCP can be maintained using either 10% ethanol or 10 mM methionine, which showed a RCP of 90.3 ± 2.2% and 89.2 ± 2.0% after 24 h, respectively. In the case of labeling performed under ‘downscaled’ therapeutical conditions (i.e., 0.063 GBq/1.5 nmol in 0.28 mL), 10% ethanol or a combination of ethanol and methionine (7% and 3.5 mM, respectively) offers the best protection to reduce radiolysis (according to the authors).

Obviously, different radionuclides, due to the decay rate, type of decay, and energy of the emitted particles, will create different dose loads in radiopharmaceutical preparations, and different vector molecules, due to their structural features, will exhibit different sensitivities to radiolysis. However, when comparing the results of various scientific studies on the stability of radiopharmaceuticals under radiolysis conditions, even within the framework of one therapeutic radionuclide (e.g., ^177^Lu) and one vector molecule already known in clinical practice (e.g., PSMA-617 or DOTA-TATE), significant discrepancies were found. These differences are due to several factors, including the use of different synthesis protocols, different concentrations of activity, different amounts of components (amount of precursor, amount of antioxidant, nature, and concentration of buffer agent), the volume of the reaction mixture, and storage temperature. At the same time, some scientific groups, comparing the effectiveness of various radioprotectors, designed their studies based on the same masses of substances, while others used the same molar concentrations. In addition, the difference in the results is due to the use of different approaches to determine the radiochemical purity of the radiopharmaceuticals. In some studies, only paper and thin-layer chromatography (TLC) methods were used, whereas others used only HPLC methods or a combination of TLC and HPLC. However, even when similar HPLC methods are used in combination with TLC, there are questions regarding the differences in the parameters of the analytical equipment, inter-laboratory validation (reproducibility), and, in general, the interpretation of the resulting radio-chromatograms [54,56]. All these differences make it extremely difficult to adequately compare the results obtained.

This work is our preliminary result of evaluating the dependencies of the radiolytic degradation of vector molecules in therapeutic radiopharmaceuticals on key preparation parameters, including activity concentration, absorbed dose generated by the radionuclide, buffer agent concentration, precursor amount, and dosage form volume. The main goal of these studies is an attempt to establish the necessary correlations for RCP prediction and the effective optimization of therapeutic radiopharmaceutical composition in terms of their resistance to radiolysis, which can be useful at the stage of pharmaceutical development. Lutetium-177 was chosen as the therapeutic radionuclide considering the above-mentioned advantages. The PSMA-617 molecule was chosen as the object of study, as it is one of the most widely known vector molecules actively used in clinical practice for radionuclide therapy worldwide. Considering the available data on the composition of different therapeutic radiopharmaceuticals, as well as published data on specific bimolecular rate constants for the reactions of different radicals with organic compounds in aqueous solutions, several compounds were selected and investigated in terms of their effectiveness in suppressing radiolysis processes in [^177^Lu]Lu-PSMA-617 preparations.

## 2. Results

### 2.1. Radiochemical Purity of [^177^Lu]Lu-PSMA-617 and Its Radiolysis-Induced Decrease

**Figure 1:**
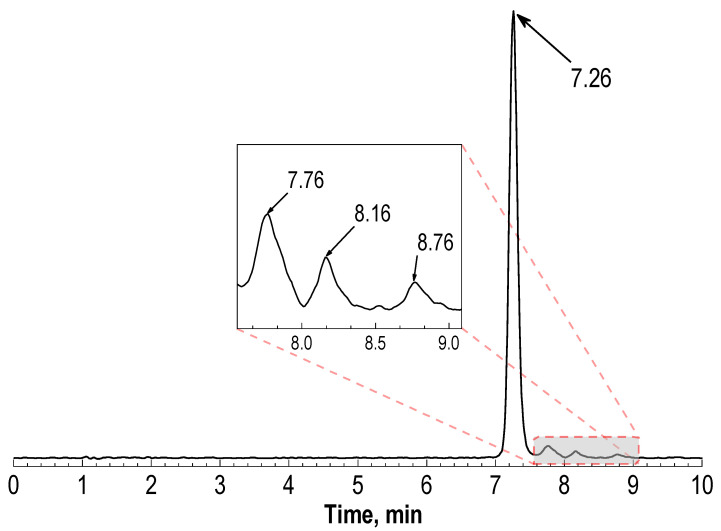
Typical radio-HPLC chromatogram of [^177^Lu]Lu-PSMA-617 preparation obtained via HPLC method 3 (see Section 4): sample volume—1 mL, ^177^Lu activity in the sample—150 MBq, 10 μg of PSMA-617, pH 4.5, synthesis at 95 °C for 30 min (for corresponding radio-TLC chromatograms, see Supplementary Materials Figure S1).

A typical radio-HPLC chromatogram of the [^177^Lu]Lu-PSMA-617 preparation is shown in Figure 1.

In addition to the main peak (Rt = 7.2 ± 0.1 min) corresponding to the [^177^Lu]Lu-PSMA-617, the chromatogram also contains three peaks of radiochemical impurities (Rt~7.8, 8.2 and 8.8 min, respectively). These radiochemical impurities were recently identified as products of structural changes in the PSMA-617 pharmacophore Glu-C(O)-Lys [37]. A comparison of the [^177^Lu]Lu-PSMA-617 chromatograms obtained using various HPLC methods, including those used by the authors of [37], is shown in Figures S2 and S3. The nascent impurities were the result of the spontaneous thermally mediated condensation of the Glu-C(O)-Lys fragment, leading to the formation of three five-membered cyclic forms. The analysis results of different [^177^Lu]Lu-PSMA-617 samples showed that the total amount of lutetium-177 activity associated with these radiochemical impurities can be ≥5% (in some cases, 10–15%). It has been shown that the formation of these by-products is mainly dependent on the synthesis temperature. Since the mechanisms and dependences of these impurities’ formation are beyond the scope of this work, all radiochemical purity (RCP) values presented herein are corrected for their content unless otherwise stated.

**Figure 2:**
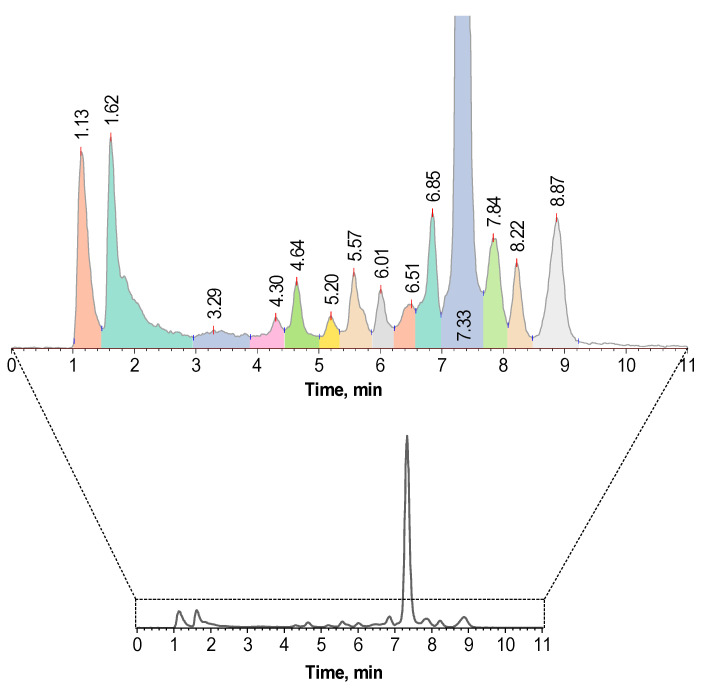
The radio-HPLC chromatogram of [^177^Lu]Lu-PSMA-617 preparation obtained with HPLC method 1 at EOS time: sample volume—1 mL, ^177^Lu activity in the sample—4.8 GBq, 100 μg of PSMA-617, pH 4.5, C(Na-acetate) = 0.03 mol/L, synthesis at 95 °C for 30 min (corresponding radio-TLC chromatograms are presented in Figure 3).

If the synthesis of [^177^Lu]Lu-PSMA-617 was carried out without any excipients and with lutetium-177 activities close to those required for clinical use, the analysis results clearly demonstrated that RCP of the final preparation is already below any possible acceptance criteria (<95%) at the end-of-synthesis (EOS) time—Figure 2.

**Figure 3:**
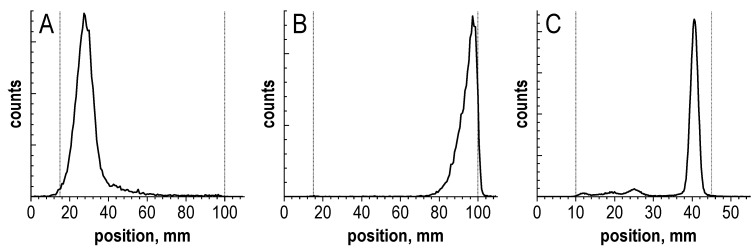
Radio-TLC chromatograms of 4.8 GBq [^177^Lu]Lu-PSMA-617 preparation (sample volume—1 mL, 100 μg of PSMA-617, pH 4.5, synthesis at 95 °C for 30 min) obtained with different TLC methods (see Section 4): (**A**) method 1 (*Rf* for [^177^Lu]Lu-PSMA-617 is 0.15 ± 0.05), (**B**) method 2 (*Rf* for [^177^Lu]Lu-PSMA-617 is 0.95 ± 0.05), (**C**) method 3 (*Rf* for [^177^Lu]Lu-PSMA-617 is 0.85 ± 0.05).

**Figure 4:**
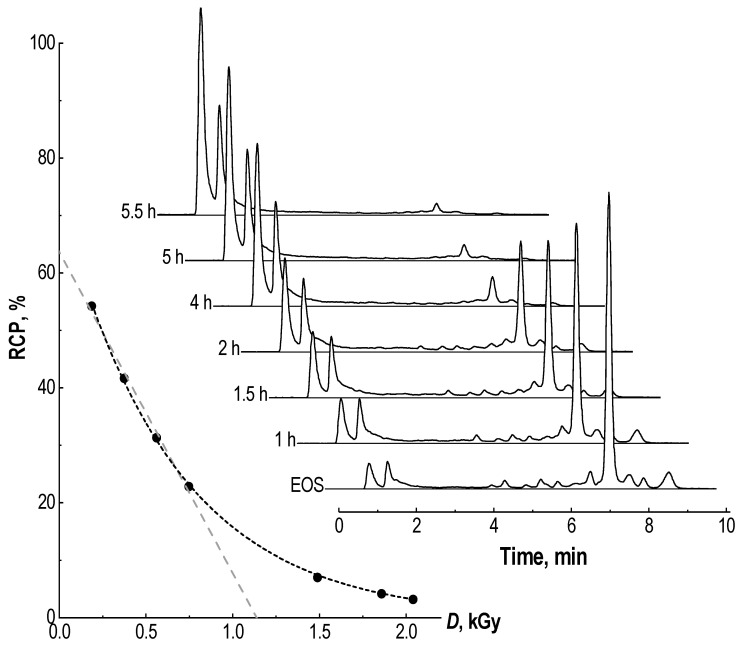
Dependence of [^177^Lu]Lu-PSMA-617 radiochemical purity on the absorbed dose (kGy) accumulated during storage (**left**) and corresponding radio-HPLC chromatograms (**right**): sample volume—1 mL, ^177^Lu activity in the sample—4.8 GBq, 100 μg of PSMA-617, pH 4.5, C(Na-acetate) = 0.03 mol/L, synthesis at 95 °C for 30 min (black dashed line here represents exponential approximation of the experimental data Y = 73·exp(−X/653), R^2^ = 0.9996; linear regression for the first four data points (gray dashed line)—Y = 64−0.056·X, R^2^ = 0.9962); for corresponding radio-TLC chromatograms see Supplementary Materials Figure S4.

In addition to the main peak of [^177^Lu]Lu-PSMA-617 as well as the above-described thermal cyclization impurities (12.2% in total), at least 10 additional side radiochemical forms could be identified in the radio-HPLC chromatogram (33.6% in total), which arose under the action of radiolysis in the preparation. With a practically quantitative level of radiochemical conversion (absence of unbound ^177^Lu—Figure 3), the radiochemical purity of the preparation at the end of the synthesis was only about 54%. This means that almost half of all ^177^Lu activity in the preparation was associated with chemical forms other than the main receptor-specific molecule. Further changes in the radiochemical purity of the [^177^Lu]Lu-PSMA-617 preparation over time (considering all detectable impurities), and related to the absorbed dose D (kGy), are shown in Figure 4.

**Figure 5:**
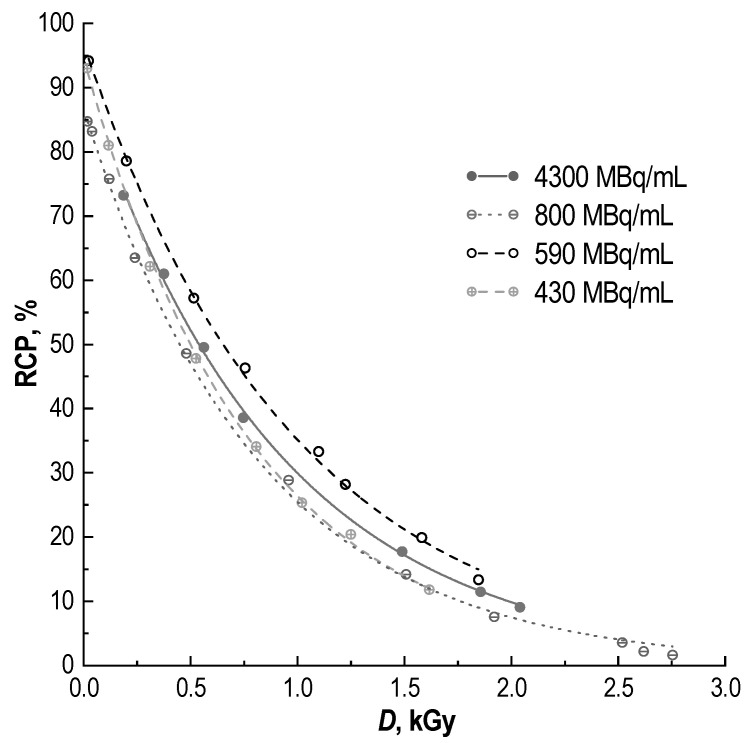
The dependence of the [^177^Lu]Lu-PSMA-617 radiochemical purity on the absorbed dose for different activity concentrations (samples volume—1 mL, 100 μg of PSMA-617, pH 4.5, C(Na-acetate) = 0.03 mol/L; error bars are omitted for clarity).

The radiochemical purity is inversely proportional to the absorbed dose in the [^177^Lu]Lu-PSMA-617 preparation and has an exponential dependence (linear regression can only be established for starting points when the absorbed dose is less than ~0.75–0.80 Gy—see Section 3). When different initial lutetium-177 activities were used in samples of the same composition, the change in their radiochemical purity over time differed significantly. However, when recalculating the storage period and activity (with decay correction) per absorbed dose in the sample, it became clear that, *ceteris paribus*, the absorbed dose was the key factor influencing the RCP value (Figure 5).

Analyzing the radio-HPLC chromatograms of the [^177^Lu]Lu-PSMA-617 preparations that underwent radiolysis over time, an interesting fact can be noted: on the chromatograms obtained by method 1, there was a significant increase in the radiochemical impurity with a retention time of 1.1 min (corresponding to unbound [^177^Lu]Lu^3+^) as well as the second impurity with a close retention time of 1.6 min. This trend was also observed for other HPLC methods (see Supplementary Materials, Figures S2 and S3). At the same time, the TLC analysis data did not confirm the content of unbound [^177^Lu]Lu^3+^ impurities in the samples (or at least not in such quantities; see Figure 3 and Supplementary Materials, Figure S4). This difference cannot be substantiated by the non-specific sorption of unbound [^177^Lu]Lu^3+^ on the HPLC column [43,57,58,59] because, in this particular case, the values determined by the HPLC methods significantly exceed the values obtained by the TLC methods. This has also been observed in several other studies [45,46]. To confirm that this impurity is not a cationic form of ^177^Lu or its weak associate with other possible ligands in the sample (such as acetate-anion), we conducted a comparative experiment using a CM cation-exchange cartridge (Sep-Pak Accell Plus CM Plus Light Cartridge (Milford, MA, USA)), which is often used for the final purification of radiopharmaceuticals with metal radionuclides in manual and automated synthesis. Summarily, [^177^Lu]Lu^3+^ was added to a [^177^Lu]Lu-PSMA-617 sample (synthesized with a high RCP (>98%)) in the form of a solution identical to the composition of the preparation, but without a precursor (150 MBq of ^177^Lu, pH 4.5, C(Na-acetate) = 0.03 mol/L). As a reference sample, the [^177^Lu]Lu-PSMA-617 preparation obtained with an RCP of >98%, which underwent radiolysis during storage, was used. Both samples were passed through CM cartridges. Radio-HPLC analysis was performed before and after the contact of the samples with the cation-exchange resin (see Supplementary Materials Figure S5). The sample with the addition of unbound [^177^Lu]Lu^3+^, after passing through the cartridge, almost returned to its original RCP value (content of impurities with a retention time of 1.1–1.6 min was 17.2 + 1.1% and 1.5 + 0.5% before and after contact with CM cartridge, respectively). No significant difference in the RCP value was found for the sample that underwent radiolysis after contact with the cation-exchanger: the content of impurities with a retention time of 1.1–1.6 min was 10.1 + 17.1% and 10.1 + 17.6% before and after contact, respectively. Therefore, these impurities were not associated with the release of lutetium-177 from the chelator ring. If this impurity were a ^177^Lu-associated chelator detached from the [^177^Lu]Lu-PSMA-617 molecule due to radiolysis, then a clear correlation would also be observed between the TLC and HPLC data [60,61]. This requires a separate detailed study of the nascent impurity nature (possibly using the HPLC-MS method), but it must be considered when determining the radiochemical purity of ^177^Lu-radiopharmaceuticals with high activity concentrations, as it can distort the real picture of the labeling efficiency and radiochemical conversion.

### 2.2. Effect of Precursor Amount and Sample Dilution

**Figure 6:**
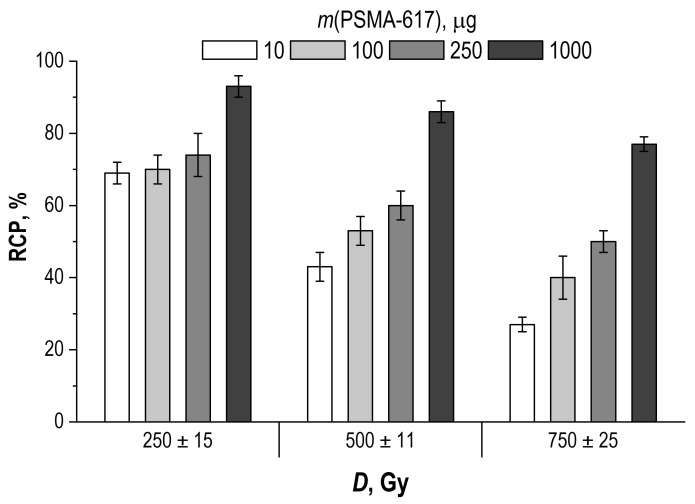
Influence of PSMA-617 precursor amount in the sample on the degree of radiolitic degradation of [^177^Lu]Lu-PSMA-617 at different absorbed doses (samples volume—1 mL, ^177^Lu activity in the sample—450 MBq, pH 4.5, C(Na-acetate) = 0.03 mol/L; RCP value is presented as mean ± SD, *n* = 3).

The results of assessing the effect of the precursor amount on the degree of radiolytic degradation at different absorbed doses are presented in Figure 6.

As can be seen from the experimental data, an increase in the amount of the precursor can increase the lifetime of the radiopharmaceutical. This parameter also often affects the yield of the labeling reaction (in the direction of the increase). However, in the case of receptor-specific radiopharmaceuticals, the value of specific (molar) activity is an overriding factor, and the amount of precursor must be chosen primarily based on this criterion. The influence of the precursor amount on the radiation stability is a secondary factor when choosing the composition of the finished dosage form, but it must be considered when comparing experimental data from different studies.

**Figure 7:**
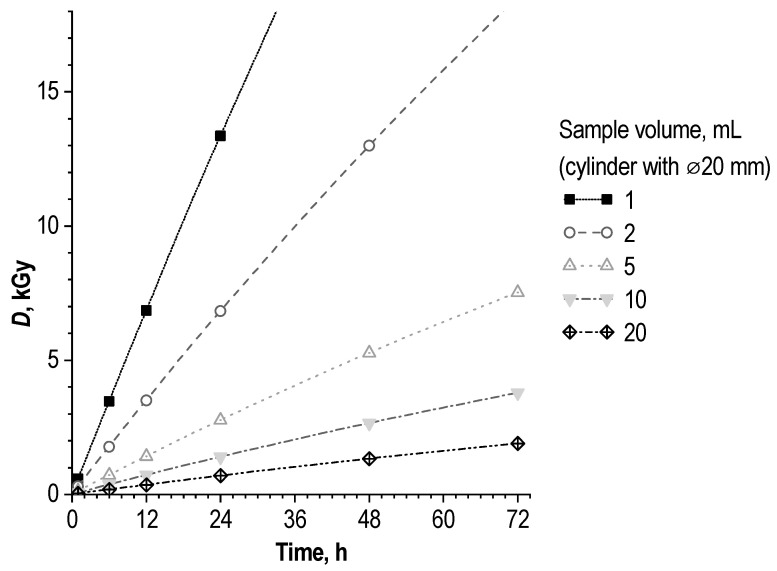
Dependence of absorbed dose growth over time on the volume of the sample (for 7.4 GBq of ^177^Lu, aqueous solution in the geometry of a cylinder with a diameter of 20 mm, Gent4 simulation).

Based on the results of computer modeling (Geant4 with PENELOPE physics list) for an aqueous solution in the geometry of a cylinder with a diameter of 20 mm (simulating the geometry of a liquid in a standard injection vial ISO 8362-1:2018), as well as Fricke chemical dosimetry [62], the absorbed dose value over time significantly depends on the sample volume (volume of the cylinder)—Figure 7.

**Figure 8:**
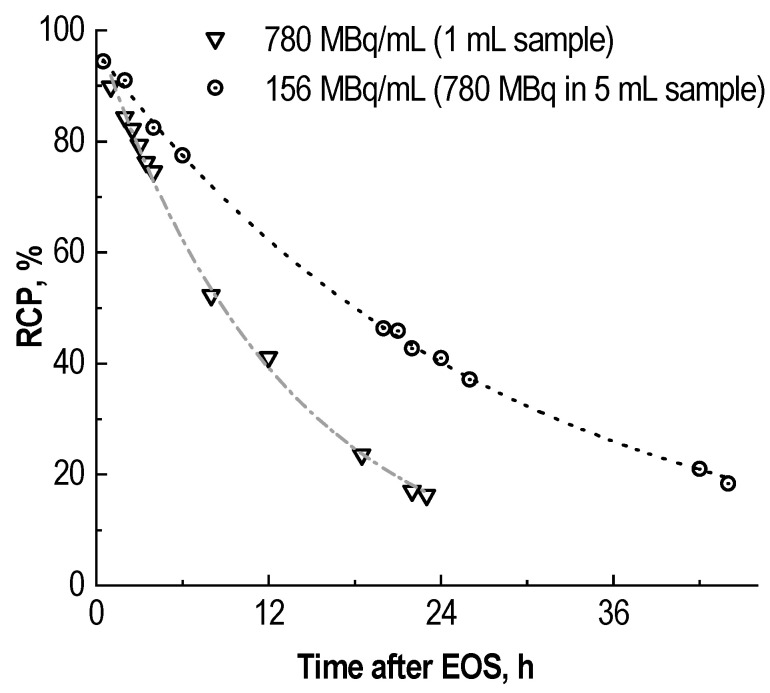
Comparison of radiochemical purity decrease over time for two [^177^Lu]Lu-PSMA-617 preparations: 1 mL sample with 780 MBq of ^177^Lu and identical sample diluted with water (×5); synthesis at 95 °C for 30 min in 1 mL, 100 μg of PSMA-617, pH 4.5, C(Na-acetate) = 0.03 mol/L (mean RCP value, *n* = 3).

The results of comparing the radiolytic degradation of two [^177^Lu]Lu-PSMA-617 preparations with the same total activity but different volumes corresponded to the absorbed dose estimation—Figure 8. The radiochemical purity of the [^177^Lu]Lu-PSMA-617 preparation with a higher activity concentration (780 MBq in 1 mL) after 24 h of storage was 16.8 ± 3.3%, while of the preparation with a lower activity concentration (780 MBq in 5 mL) was 40.1 ± 2.7%.

Hence, an increase in the volume of the final dosage form (dilution of the activity concentration) within reasonable limits helps lengthen the shelf-life of ^177^Lu–radiopharmaceuticals. This approach is especially justified if it is acceptable to convert an injectable dosage form into an infusion, but itself cannot provide sufficient stability for radiopharmaceutical preparation. An increase in the volume of the final dosage form can be used only as an auxiliary condition (as well as to lower the storage temperature [38]).

### 2.3. Effect of Buffering Agent (Sodium Acetate) Concentration

**Figure 9:**
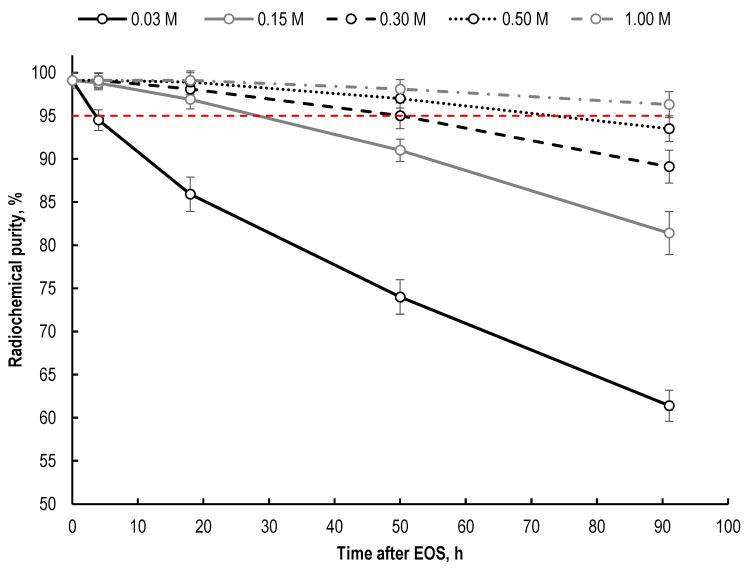
Time dependences of the decrease in [^177^Lu]Lu-PSMA-617 radiochemical purity on the concentration of sodium acetate in the final preparation (sample volume—1 mL, ^177^Lu activity in each sample—100 MBq, 100 μg of PSMA-617, pH 4.5; RCP value is presented as mean ± SD, *n* = 3).

The dependence of [^177^Lu]Lu-PSMA-617 radiolytic degradation on sodium acetate concentration in the preparations is shown in Figure 9.

**Figure 10:**
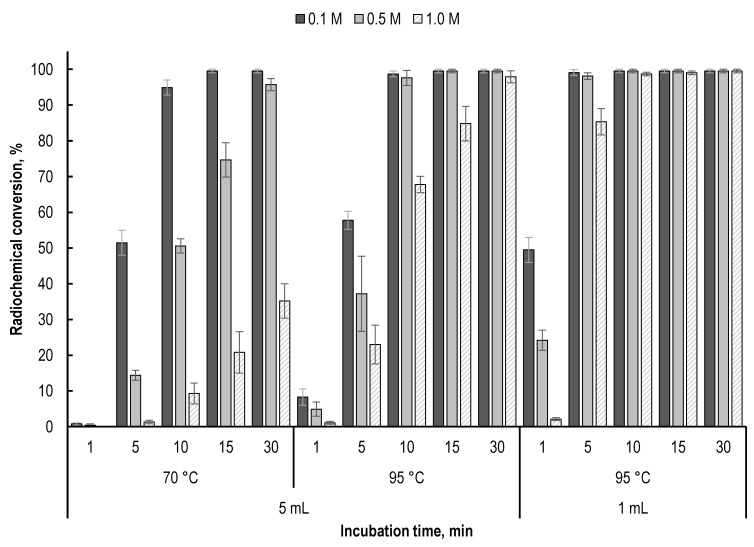
Influence of sodium acetate concentration at different temperatures and volumes of the reaction mixture on the kinetics of [^177^Lu]Lu-PSMA-617 formation (^177^Lu activity in each sample—150 MBq, 20 μg of PSMA-617, pH 4.5; labelling yield (radiochemical conversion) value is presented as mean ± SD, *n* = 3).

Although acetate ions are very weak free-radical scavengers, an increase in their concentration in the sample can suppress radioconjugate degradation [27]. If the acidity of the preparation is provided solely by the hydrochloric acid of lutetium-177 solution (0.04–0.05 M HCl), then the maximum concentration of sodium acetate (when the required pH level is reached) is only about 0.08–0.1 M. In the case of using an acetate buffer solution or the presence of additional acid additives in the sample (such as gentisic or ascorbic acids), the final concentration of sodium acetate can be significantly increased. It should be noted that an increase in the concentration of sodium acetate can affect the kinetics of lutetium-177 incorporation into the PSMA-617 structure; however, this effect becomes noticeable only at low synthesis temperatures and/or large volumes of the reaction mixture (Figure 10).

However, even at high concentrations of sodium acetate in the final preparation (1 mol/L), the radiolytic degradation of [^177^Lu]Lu-PSMA-617 occurs extremely quickly at a lutetium-177 activity close to that required for clinical use: in the [^177^Lu]Lu-PSMA-617 preparation with 5 GBq of ^177^Lu (sample volume—1 mL, 100 μg of PSMA-617, pH 4.5, C(Na-acetate) = 1 mol/L), the RCP value decreases from 97% (at EOS) to 82.6%, 69.7%, 57.4% and 43.2% after 1, 2, 3 and 6 h of storage, respectively (see Supplementary Materials Figure S6). Thus, the amount of sodium acetate in the dosage form of lutetium-177 RPs (e.g., [^177^Lu]Lu-PSMA-617) may be optimized (increased) to further extend its radiation stability; however, the need for the additional use of appropriate quenchers is beyond doubt. In addition, the influence of the concentration of the buffer used in synthesis should be considered when comparing the data on the stability of the ^177^Lu–preparations obtained from different scientific groups (using different synthesis protocols).

### 2.4. Effectiveness of Different Quenching Excipients

**Figure 11:**
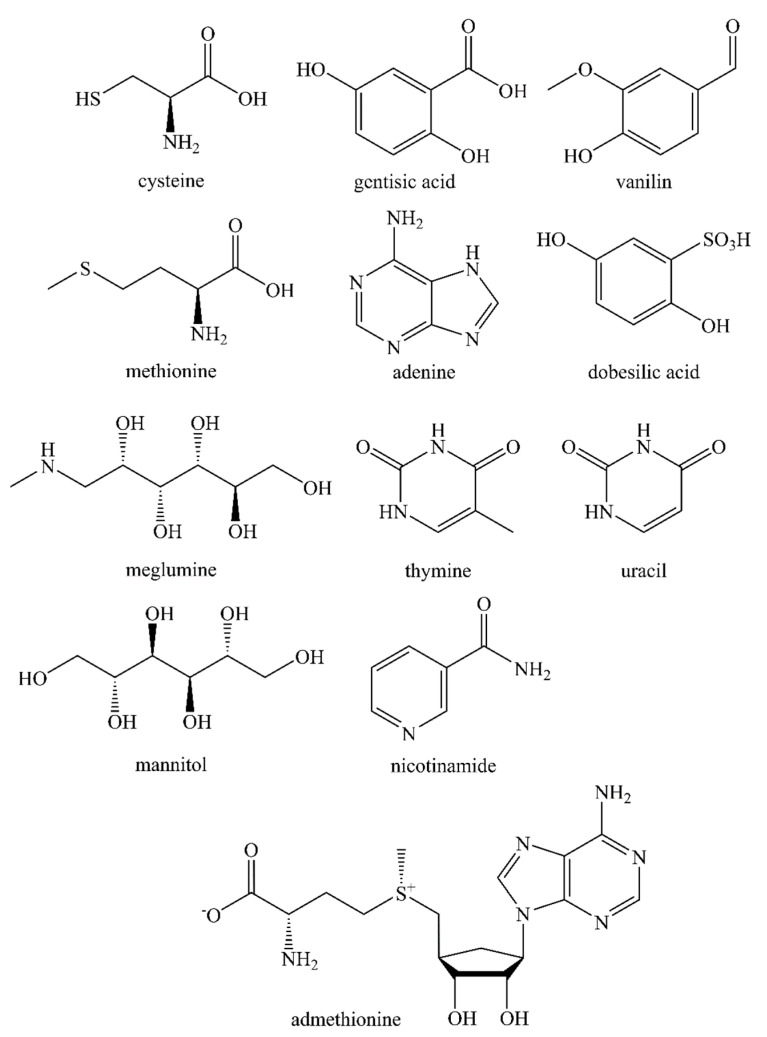
The molecules investigated as radical scavengers for [^177^Lu]Lu-PSMA-617 preparations.

The compounds studied in this work as radical scavengers to suppress radiolysis are shown in Figure 11.

**Figure 12:**
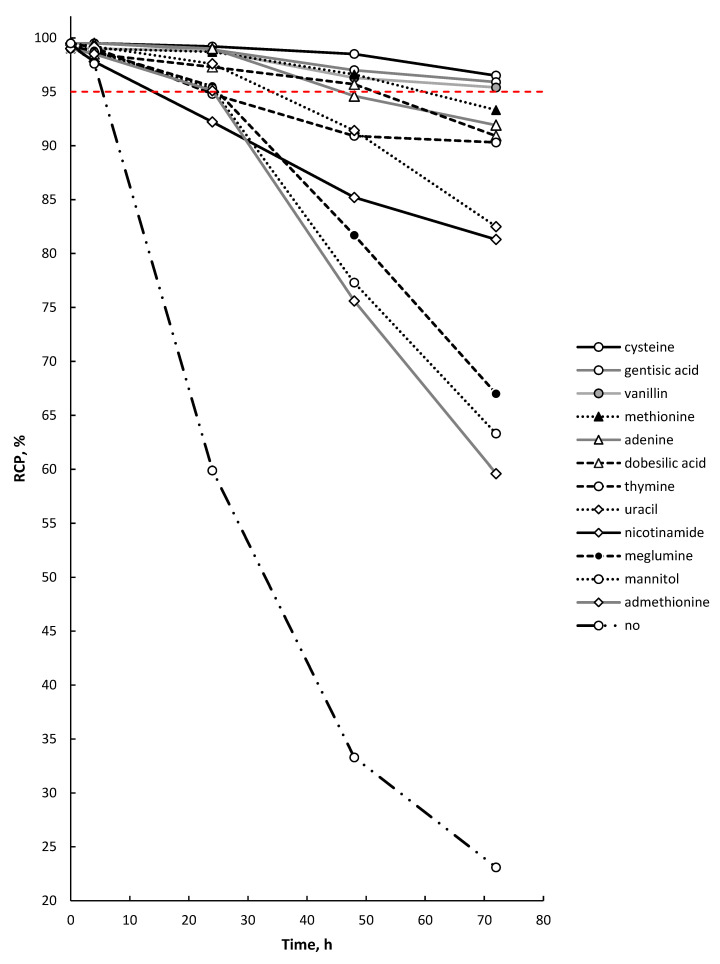
Influence of various quencher additions on the maintenance of [^177^Lu]Lu-PSMA-617 radiochemical purity over time. Samples volume—1 mL, ^177^Lu activity in the sample—450 MBq, pH 4.5, synthesis at 95 °C for 30 min, 100 μg of PSMA-617 C(Na-acetate) = 0.03 mol/L (RCP value is presented as mean, *n* = 3; error bars are omitted for clarity).

The molar concentration of all the quenching excipients used was kept the same (7.4 μM/mL) and was determined based on the maximum solubility in water of the least soluble compound (adenine, 1.003 mg/mL). None of the substances studied (in the indicated quantities) had any significant effect on the degree of lutetium-177 radiochemical conversion during [^177^Lu]Lu-PSMA-617 synthesis. A comparison of the effectiveness of various quenchers against the radiolytic degradation of [^177^Lu]Lu-PSMA-617 is presented in Figure 12.

**Figure 13:**
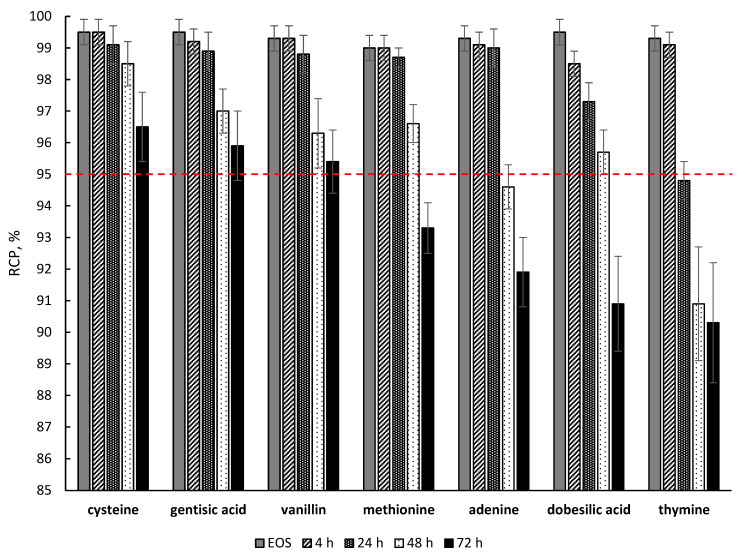
Influence of selected quenchers on the maintenance of [^177^Lu]Lu-PSMA-617 radiochemical purity over time. Samples volume—1 mL, ^177^Lu activity in the sample—450 MBq, pH 4.5, synthesis at 95 °C for 30 min, C(Na-acetate) = 0.03 mol/L (RCP value is presented as mean ± SD, *n* = 3).

More detailed data for the seven most effective compounds are presented in Figure 13.

Among all the studied compounds, cysteine, gentisic acid, and vanillin showed the best radioprotective function in relation to the [^177^Lu]Lu-PSMA-617 molecule (with a statistically significant difference of *p* < 0.05 compared to the next substance in order—methionine—and *p* < 0.01 compared to all other studied substances) after 72 h of storage. It is noteworthy that all three substances individually showed almost the same RCP value for [^177^Lu]Lu-PSMA-617 after 72 h of storage (96.5 ± 1.0%, 95.9 ± 1.1% and 95.4 ± 0.9% for cysteine, gentisic acid and vanillin, respectively (*p* > 0.05)). When comparing the effectiveness of quenchers at earlier time points (48 h after EOS), cysteine was found to be the most effective substance (98.6 ± 0.7%, 97.0 ± 0.6% and 96.3 ± 1.1% for cysteine, gentisic acid and vanillin, respectively).

**Figure 14:**
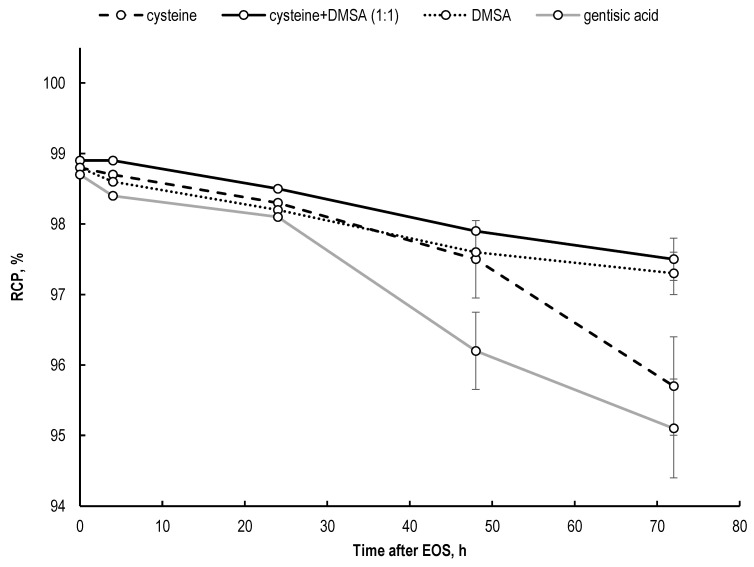
Influence of the addition of various quenchers on the maintenance of [^177^Lu]Lu-PSMA-617 radiochemical purity over time. Samples volume—1 mL, ^177^Lu activity in the sample—470 MBq, 100 μg of PSMA-617, pH 4.5, C(Na-acetate) = 0.03 mol/L (RCP value is presented as mean ± SD, *n* = 3).

For all the attractiveness of cysteine as effective scavenger of free radicals [63,64,65,66,67], one undesirable fact was noted during the experiments: in the course of storing the cysteine-containing samples, insoluble particles began to appear as an albescent powdery deposit on the walls of the vial or as a well-defined whitish precipitate at the bottom of the vial (depending on the lutetium-177 activity used and the storage time). This precipitate was identified as cystine [68], and its formation is associated with the radiation-induced oxidation of cysteine [69,70]. In itself, this fact is not surprising, since exactly due to the cystine formation, cysteine itself has high specific bimolecular rate constants (k, L·mol^−1^·s^−1^) for interaction with radicals (for example, in the case of H· radical, k = 1.0 × 10^9^ (pH 6) for cystine formation, and k = 1.2 × 10^8^ (pH 6) for H_2_S formation [71]). However, it was unexpected that the formation of cystine occurred so quickly, and in such quantities that the insoluble precipitate became visible to the naked eye within a short storage time. The formation of insoluble particles is unacceptable for injectable dosage forms (except for radiopharmaceuticals where the radiocolloids themselves are the main active form). We attempted to minimize the negative effects of cystine formation while retaining the properties of cysteine as a radical scavenger. For this purpose, we evaluated the effect of the complementary addition of DMSA to ^177^Lu–preparations containing cysteine. *meso*-Dimercaptosuccinic acid (DMSA) is a metal-binding agent that has been shown to be effective in the treatment of lead, mercury, and arsenic intoxication. When radiolabeled with technetium-99m ([^99m^Tc]Tc-DMSA), it is used for SPECT renal imaging [72,73,74]. However, the most important thing is that DMSA was proposed as a potential therapy for cystinuria [75,76] because of its ability to react with cysteine (and cystine) and form a highly soluble mixed disulfide [77,78] (the proposed pathway for the formation of the major mixed disulfide of DMSA with L-cysteine is presented in Figure S7 (Supplementary Materials)). No negative effect of the addition of DMSA to the reaction mixture on [^177^Lu]Lu-PSMA-617 synthesis was observed during the experiments. Interestingly, DMSA itself was found to be an effective quencher for ^177^Lu-preparations (Figure 14). DMSA and its mixture with cysteine showed a greater radioprotective function (*p* < 0.01) in relation to the [^177^Lu]Lu-PSMA-617 degradation than cysteine and gentisic acid separately (after 72 h of storage, RCP was 97.3 ± 0.3%, 97.5 ± 0.3%, 95.7 ± 0.7% and 95.1 ± 0.8% for DMSA, DMSA + cysteine (1:1), cysteine, and gentisic acid, respectively).

No statistically significant difference was observed in the maintenance of [^177^Lu]Lu-PSMA-617 radiochemical purity when cysteine and DMSA were used in 1:1 and 1:½ molar ratios (in accordance with the proposed pathway for the formation of mixed disulfide)—Figure S8, Supplementary Materials.

**Figure 15:**
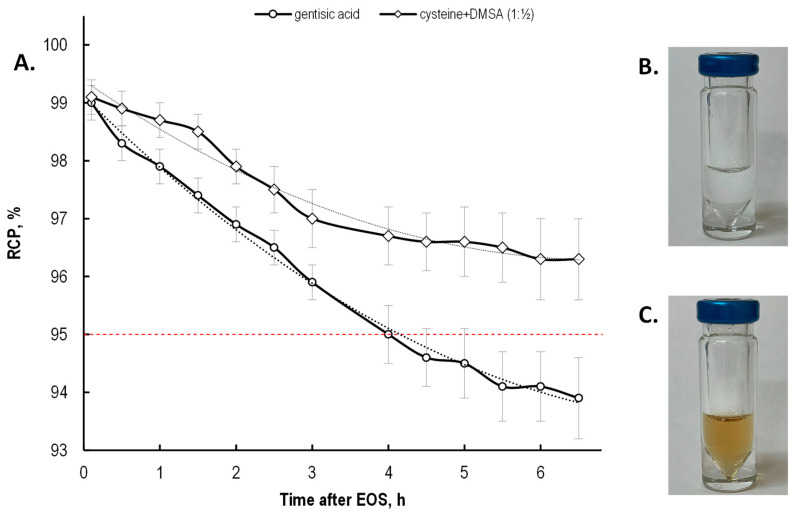
(**A**) Change in the radiochemical purity of [^177^Lu]Lu-PSMA-617 preparations with 7.4 GBq of ^177^Lu (samples volume—2 mL, pH 4.5, synthesis at 95 °C for 30 min, C(Na-acetate) = 0.15 mol/L, gentisic acid—5 mg (32 μmol), cysteine—3.9 mg (32 μmol); RCP value is presented as mean ± SD, *n* = 3); (**B**) Photograph of the 7.4 GBq [^177^Lu]Lu-PSMA-617 sample prepared with cysteine 3.9 mg (32 μmol) + DMSA 2.9 mg (16 μmol) after 24 h of storage at RT; (**C**) Photograph of the 7.4 GBq [^177^Lu]Lu-PSMA-617 sample prepared with 5 mg gentisic acid (32 μmol) after 24 h of storage at RT.

Based on the obtained results, in the final part of this study, we compared the effectiveness of a gentisic acid and cysteine + DMSA mixture in relation to [^177^Lu]Lu-PSMA-617 preparations with the clinically required activity of lutetium-177—Figure 15. According to published data, when gentisic acid was used alone as a stabilizer during the synthesis of ^177^Lu-PSMA radiopharmaceuticals with the clinical activity of ^177^Lu, its amount was 5–10 mg per sample [39,40]. Hence, the comparison was carried out for [^177^Lu]Lu-PSMA-617 preparations (7.4 GBq of ^177^Lu), one of which contained gentisic acid (5 mg, 32 μmol), and the other containing cysteine (3.9 mg, 32 μmol) + DMSA (2.9 mg, 16 μmol). 

After 6.5 h of storage at room temperature, the ‘cysteine + DMSA’ sample showed a higher RCP than the ‘gentisic acid’ sample (96.3 ± 0.6% and 93.9 ± 0.7%, respectively; *p* < 0.02). Notably, the [^177^Lu]Lu-PSMA-617 samples already obtained with gentisic acid during the first day after synthesis began to acquire a brown color (which became more saturated during further storage—Figure 15C). This color change is most likely due to the formation of gentisic acid oxidation products (such as 1,4-benzoquinone-2-carboxylic acid and others [46,48,79]) upon interaction with radicals during radiolysis. The samples prepared with the mixture of cysteine and DMSA remained clear and colorless throughout the monitoring period (up to 144 h), and no eye-visible precipitate formation was observed.

## 3. Discussion

The main factor limiting the lifetime of diagnostic radiopharmaceuticals (especially those based on short-lived radionuclides such as ^15^O (2.03 min), ^13^N (9.97 min), ^11^C (20.3 min), ^18^F (109.8 min) and ^68^Ga (67.7 min)), is the decay of a radionuclide, resulting in a decrease in activity below the level required for clinical use. In the case of therapeutic radiopharmaceuticals based on β^−^– and α–emitting radionuclides, which have significantly longer half-lives (e.g., ^177^Lu (6.6 d), ^153^Sm (46.3 h), ^90^Y (2.7 d), ^131^I (8 d), ^225^Ac (9.9 d), etc.), the most significant limitation on the lifetime is dosage form stability and an ability to maintain the established properties under the action of radiolysis.

De Blois et al., using the ^111^In/^177^Lu-labelled compounds DOTA-bombesin and DOTA-MG11, found a linear correlation between the RCP (%) and absorbed dose (Gy) in preparations [52]. This dependence was proposed to predict the RCP of radiopeptides, in accordance with Monte Carlo calculations [53,59]. However, these results are not consistent with those of our work. Indeed, the absorbed dose is the key factor influencing the value of RCP in radiopharmaceutical preparations. In [52], the estimated dose range was 0–250 Gy. According to our results (Figure 4 and Figure 5), linear approximation is admissible only at absorbed doses of up to 0.75–0.80 kGy. At the same time, the Monte Carlo simulation results, together with chemical dosimetry (Figure 7), demonstrate that in the case of lutetium-177 clinical activity (7.4 GBq), an absorbed dose of 1 kGy will be formed after 1.71, 3.36, 8.37, 16.86 and 35.27 h of storage in preparations of 1, 2, 5, 10 and 20 mL, respectively.

According to our experimental data, the radiochemical purity has an exponential dependence on the absorbed dose (which is in better agreement with the law of radioactive decay) and is generally expressed by the empirical equation:

(2) $$\mathrm{RCP}={\mathrm{RCP}}_{0}\cdot e^{-D\kappa }$$

where RCP is the radiochemical purity (%); D is the absorbed dose (Gy) formed up to time t (h); and κ is the dose constant (Gy^−1^) [80,81] at a given amount of precursor (i.e., PSMA-617) in 1 mL.

The absorbed dose D (Gy) can be estimated using the following equation:

(3) $$D=\frac{\Delta A}{\lambda }\cdot \frac{\sum_{i}E_{i}y_{i}\varphi_{i}}{M}$$

where M is the mass of the solution (kg); y_i_—the probability of emission of the i-th particle with energy E_i_ (J/dis); λ is the radionuclide decay constant (s^−1^); ${\Delta A=A}_{0}{(1\;-\;e}^{-\lambda t})$ is the decrease in the initial activity A_0_ (Bq) of a radionuclide after time t (s); and φ_i_ is the energy fraction of the i-th particle absorbed by the solution [82].

The second factor in Equation (3) (the sum $\underset{i}{\sum }E_{i}y_{i}\varphi_{i}$ divided by the mass of the solution) is a certain coefficient that is constant for a given volume of the solution, denoted in some articles as *S* (Gy/dis or Gy·Bq^−1^·s^−1^) and mainly evaluated in silico [52,53,83]. For convenience, this coefficient can be expressed in units often used in practice (Gy·mL·MBq^−1^):

(4) $$\psi =\frac{\sum_{i}E_{i}y_{i}\varphi_{i}}{\rho \lambda }$$

wherein *S* = ψ·10^−6^·λV^−1^. Then, (3) can be written as:

(5) $$D=\frac{\Delta A}{V}\cdot \psi$$

If the activity concentration is measured in MBq/mL, then the dose coefficient ψ represents the absorbed dose that is formed in a unit of solution volume (1 mL) with a density ρ (kg/mL) during the decay of 1 MBq of radionuclide. Equation (5) does not explicitly contain geometric parameters (for example, the height of a liquid cylinder *h* and its radius *r*) but is a complex function: ψ = *f*{ρ,φ*_i_*(ρ,*E_i_*,*r*,*h*…)}. This makes it possible to determine the absorbed dose for any activity concentration when determining the dependence of ψ on geometric factors.

**Figure 16:**
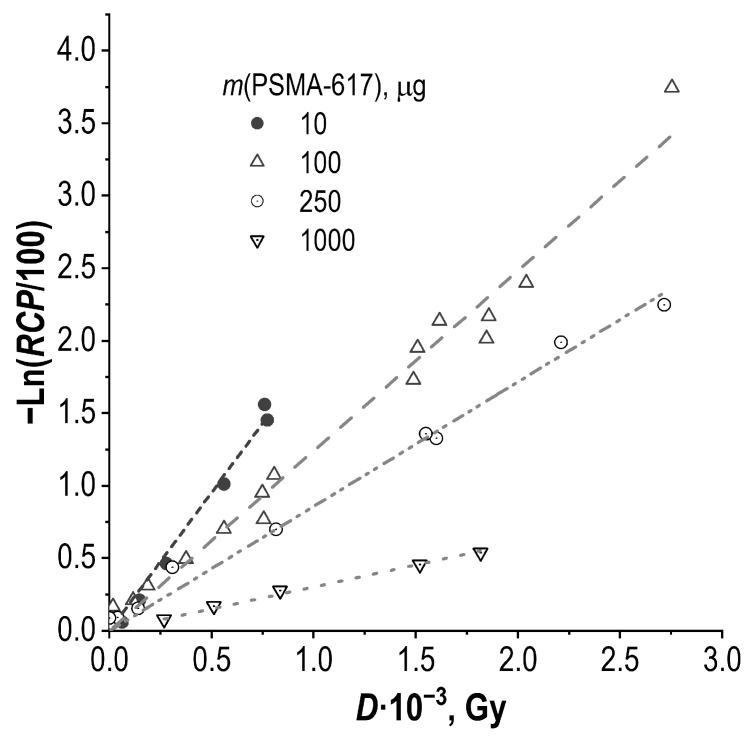
Influence of PSMA-617 precursor amount in the sample on the degree of radiolitic degradation of [^177^Lu]Lu-PSMA-617 during storage (samples volume—1 mL, ^177^Lu activity in the sample—450–4500 MBq, pH 4.5, C(Na-acetate) = 0.03 mol/L; error bars are omitted for clarity). Here, (κ ± *SD*)·10^3^ is: 1.911 ± 0.061 for 10 μg/mL (*R*^2^ 0.9950); 1.241 ± 0.027 for 100 μg/mL (*R*^2^ 0.9924); 0.859 ± 0.020 for 250 μg/mL (*R*^2^ 0.9952), and 0.3033 ± 0.0062 for 1000 μg/mL (*R*^2^ 0.9983).

According to the experimental data (Figure 6), the initial concentration of the precursor contributed to the degree of radiolytic degradation of the sample, depending on the absorbed dose. The decrease in the radiochemical purity from the initial value, depending on the absorbed dose for each initial concentration of the precursor, can be expressed in the form of a linear dependence, −ln(*RCP*/100) = κ*_m_*·*D*, as shown in Figure 16.

The relationship between the dose constant κ and the initial PSMA-617 concentrations C_0_ (mol/L) can be expressed as a linear function κ = p + q·lnC_0_. Thus, in general, the dependence of the radiochemical purity value on time (during which a certain absorbed dose is formed), with a given initial activity concentration and amount of the precursor, can be expressed by the empirical equation:

(6) $$\mathrm{RCP}={\mathrm{RCP}}_{0}\cdot e^{-D\cdot \kappa }={\mathrm{RCP}}_{0}\cdot e^{-\psi \cdot \frac{\Delta A}{V}\cdot \left(1-e^{-\lambda t}\right)\cdot \left[p+q\cdot \ln C_{0}\right]}$$

It should be emphasized that Equation (6) is empirical, obtained based on experimental data for a specific [^177^Lu]Lu-PSMA-617 molecule in aqueous media with a given concentration of excipients (acetate buffer) and pH value. The refinement of this equation for additional parameters of the dosage form should be addressed in further studies.

In general, the obtained data on the effectiveness of the studied compounds as radical scavengers correlate well with the published data on the specific bimolecular rate constants for the reactions of different radicals with these compounds in aqueous media [48,71,84,85,86] (see Supplementary Materials, Table S1). For example, if we focus on the ^•^OH radicals, then cysteine and gentisic acid, having constants of 3.5–4.7 × 10^10^ and 1.1 × 10^10^ (pH 7), respectively, showed the best stabilizing properties. The rest of the studied compounds are arranged in the following order in terms of the published specific bimolecular rate constants: methionine (5.1–8.5 × 10^9^), thymine (3.1–6.4 × 10^9^), uracil (3.1 × 10^9^), nicotinamide (1.4 × 10^9^). This order was in good agreement with the experimental data (Figure 12). For vanillin (which showed similar stabilizing efficacy to cysteine and gentisic acid), a constant of 3.3 × 10^9^ (pH 9) for ^•^OH radicals has been published; however, there are not much available data on this substance, which should be clarified. The results obtained for DMSA as a radical scavenger in ^177^Lu-preparations and as a cysteine stabilizer are also very interesting. Lungu et al. presented the results of radiolabeling meso-2,3-DMSA with ^177^Lu [87]. The authors reported a ^177^Lu-DMSA complex formation of approximately 62% at pH 5, and 98% at pH 9. This caused serious concerns regarding DMSA addition during the synthesis of lutetium-177 preparations. Notwithstanding this, no negative effect of DMSA on [^177^Lu]Lu-PSMA-617 synthesis was observed during our experiments. Despite a significant improvement in [^177^Lu]Lu-PSMA-617 stability when using a mixture of cysteine and DMSA, this approach still cannot be considered universal. Obviously, the use of DMSA is impossible in the case of radiopharmaceuticals, where there is a disulfide bond in the structure of the vector molecule (such as DOTA-TOC/TATE/NOC). At the same time, these results are very encouraging, since they support the further development of cysteine derivatives as stabilizers for radiopharmaceuticals (having greater solubility in the case of dimerization, such as N-acetyl-L-cysteine, L-alanyl-L-cysteine, etc.). It is important to note that the use of cysteine derivatives excludes the possibility of phenyl radical and benzoquinone derivative formation during radiolysis, in contrast to carboxybenzene derivatives (as gentisic acid).

## 4. Materials and Methods

### 4.1. Chemicals and Reagents

Only 18.2 MΩ·cm deionized water (Milli-Q Millipore, Merck, Darmstadt, Germany) was used. All chemicals and solvents used in the synthesis were of high purity or of pharmaceutical grade. The chemicals were purchased from Sigma-Aldrich/Merck (St. Louis, MO, USA) or Panreac Quimica (Barcelona, Spain) unless otherwise indicated. All solvents used for the HPLC analysis were of HPLC/gradient grade. The precursor PSMA-617 was purchased from ABX advanced biochemical compounds GmbH (Radeberg, Germany) and were also kindly provided by the Center of Molecular Research (Moscow, Russia).

### 4.2. Lutetium-177

No-carrier-added lutetium-177 as a [^177^Lu]LuCl_3_ solution in 0.04 M hydrochloric acid with the activity of 49.3 GBq/mL and the specific activity of at least 1850 GBq/mg was purchased from RADIOPREPARAT State Enterprise (Tashkent, Republic of Uzbekistan).

### 4.3. Synthesis of [^177^Lu]Lu-PSMA-617 Preparations

An aliquot of 10—100 μL of PSMA-617 aqueous solution (1 mg/mL) was mixed with 2 M sodium acetate (≥15 μL), the selected radioprotector solution in water, and [^177^Lu]LuCl_3_ (100–7400 MBq). The final concentration of each radioprotector used was 7.4 μmol/mL. The final concentration of sodium acetate was 0.03 M (unless otherwise specified). All samples had a pH level of 4.5 ± 0.1 (adjusted by 0.04 M HCl). The activity was monitored with an ISOMED 2010 dose calibrator («MED Nuklear-Medizintechnik Dresden GmbH», Dresden, Germany). Unless otherwise stated, the mixtures were incubated for 30 min at 95 °C. No further C18 purification of samples or any additional manipulations for the reformulation of samples after synthesis were carried out (except for specific experiment with dilution of activity—Figure 8).

### 4.4. TLC and HPLC Analysis

Several radio-TLC methods were used to analyze the radiochemical conversion and radiochemical purity of [^177^Lu]Lu-PSMA-617 preparations. The main TLC methods used are listed in Table 1.

Radiography of the TLC-strips was performed using a miniGita radio-TLC scanner (Elysia-Raytest, Straubenhardt, Germany) as well as a Cyclone^®^ Plus storage phosphor system (PerkinElmer, Waltham, MA, USA).

HPLC analysis of the [^177^Lu]Lu-PSMA-617 preparations was carried out using an LC-20AD Prominence chromatograph (Shimadzu Co., Ltd., Kyoto, Japan) equipped with a diode array detector SPD-M20A and a MiniScanPRO TLC/HPLC Flow-Count system (Eckert & Ziegler Eurotope GmbH, Berlin, Germany) with FC-3200 NaI/PMT and FC-3600 Plastic Scintillator/PMT-based detectors (⌀0.25 × 5 mm flow cell (PEEK tubing). Three different methods were used in this study—Table 2. The reversed phase C_18_ columns were purchased from Phenomenex^®^ (Torrance, CA, USA). In all three methods, solvent A was 0.1% (*v*/*v*) TFA in water (TFA—trifluoroacetic acid HPLC grade Sigma-Aldrich/Merck (St. Louis, MO, USA)).

### 4.5. Absorbed Dose Estimation

The self-absorbed dose was estimated using the Geant4 program [88] equipped with the PENELOPE set of physical processes. For each of the vial-solution system configurations, more than 10^8^ decay incidents were simulated. A modified Fricke chemical dosimetry system [89] containing ^177^Lu was also used to estimate the dose and dose rate. Briefly, an aqueous solution of FeSO_4_ and CuSO_4_ acidified with H_2_SO_4_ was purged with argon for 30 min and then mixed with an aliquot of the [^177^Lu]LuCl_3_ solution. The final concentrations were 0.001 M for ferrous sulfate, 0.01 M for copper sulfate, 0.005 M for sulfuric acid and 0.0025 to 0.05 M for hydrochloric acid. The mixtures were prepared at a final volume of 1 mL in the glass vials (inner diameter of 2 cm, ISO 8362). The volume activity of the clogged mixtures was measured immediately. The reference (intact) dosimetry solution was prepared in the same manner, in the absence of ^177^Lu. The experiment was replicated 3–5 times. The change in the Fe(III) concentration was monitored using a Cary 60 (Agilent Technologies Inc., Santa Clara, CA, USA) at 304 nm (room temperature, 22 °C) with an optical path length of 5 mm relative to the reference dosimetry solution. The measurements were performed using three samples from each mixture. The measured aliquots were returned to the mixture to minimize the influence of the sampling procedure on the absorbed dose formation. More detailed information on estimating the absorbed dose from lutetium-177 using computer modeling and chemical dosimetry can be found in our previous work [62].

## 5. Conclusions

The development of radionuclide therapy technologies and the successes achieved to date allow us to assert that in the coming years, this market segment will be increasingly strengthened in the field of managing patients with various pathologies worldwide. One can also expect a significant increase in the number of used therapeutic radiopharmaceuticals, which will have to meet strict quality criteria. An analysis of the published data on optimizing the composition of the dosage form of therapeutic radiopharmaceuticals shows that in most studies a purely empirical approach is implemented. This approach is based on the principle of whether there is a satisfactory effect (sufficient preparation stability) or not. However, recent studies have shown that it is possible to identify clear dependencies and correlations between the parameters of the therapeutic radiopharmaceutical dosage form and its stability over the required period. Notably, the development of the finished dosage form requires the implementation of approaches not only from radiochemistry, but also from radiation chemistry, since it is the radiolysis that is the main limiting factor. Regarding therapeutic radiopharmaceuticals, we believe that, eventually, the concept of centralized production and supply of dosage forms with guaranteed quality parameters will be increasingly implemented. In turn, this requires the formulation of therapeutic radiopharmaceuticals with sufficient stability over time. Thus, the dependencies of assessing and predicting the stability of radiopharmaceuticals under radiation exposure will significantly simplify the choice of the optimal composition of the dosage form at the stage of pharmaceutical development. It seems most convenient and effective to use the dependence of radiochemical purity on the absorbed dose (rather than radionuclide activity, time, volume, etc.) as a key characteristic for further evaluations and comparisons.

Considering the number of parameters that can affect the resistance of a vector molecule to radiolysis (including the results of this work), as well as the variety of methodological approaches implemented by various scientific groups, it is necessary to develop a unified approach to assessing the antioxidant activity of substances in therapeutic radiopharmaceuticals, as it is carried out in the pharmaceutical and food industries [90,91]. The approach based on a comparison under conditions of one standard media or in comparison with one standard substance (by analogy with the well-known TRAP (total reactive antioxidant potential or total radical-trapping antioxidant parameter [92]) and the TEAC (Trolox equivalent antioxidant capacity [93]) assay) seems to be very convenient. At the same time, it is important to consider potential antioxidants not only in terms of their effectiveness in stabilizing the dosage form, but also in terms of the substances formed during the process of radical reactions and their potential toxicity. Some of the information gathered from kinetics-based approaches showed how the environment and reacting radicals influence the main chemical routes involved in the radical-scavenging activity of antioxidants. This information was also used to propose trends regarding the activity of a large set of antioxidants in different media. Hence, the specific bimolecular rate constants for the reactions of different radicals with selected compounds in aqueous solution can be used as a convenient parameter for the primary selection of compounds. However, there are still many aspects that require further investigation with respect to therapeutic radiopharmaceuticals. Some of them are related to the chemistry of nonradical oxidants, the possible pro-oxidant effects of compounds considered only as antioxidants, synergic effects, regeneration in antioxidant mixtures, etc. [94]. Computational-based strategies together with the unification of methodological approaches might provide useful information on these topics and contribute to gain deeper physicochemical insights in the R&D of therapeutic radiopharmaceuticals dosage forms.

## Figures and Tables

**Table 1:** Radio-TLC methods used to analyze the radiochemical conversion of [^177^Lu]Lu-PSMA-617 preparations.

#	TLC Plates	Solvent	*Rf* of ^177^Lu Species		
			Unbound [^177^Lu]Lu^3+^	[^177^Lu]Lu-PSMA-617	[^177^Lu]Lu-PSMA-617 Degradation Products
1	iTLC-SG glass microfiber chromatography paper impregnated with a silica gel (Agilent, Santa Clara, CA, USA)	0.05 M H_3_Citr_aq_.	0.95 ± 0.05	0.15 ± 0.05	0.2–1.0
2		NH_3_-Ethanol-H_2_O (1:5:10)	0–0.05	0.95 ± 0.05	0.95 ± 0.05
3	TLC Silica gel 60 sheets with aluminum support (5553, Merck, Darmstadt, Germany)	MeCN-H_2_O (1:1)	0–0.05	0.85 ± 0.05	0.15–0.75
4	TLC Cellulose F sheets with plastic support (5565, Merck, Darmstadt, Germany)		0–0.05	0.95 ± 0.05	0.95 ± 0.05

**Table 2:** Radio-HPLC methods used to analyze the radiochemical purity of [^177^Lu]Lu-PSMA-617 preparations.

#	HPLC Column	Gradient Profile, Flow Rate, Solvents	*Rt* of ^177^Lu Species			
			Unbound [^177^Lu]Lu^3+^	[^177^Lu]Lu-PSMA-617	[^177^Lu]Lu-PSMA-617 Degradation Products	[^177^Lu]Lu-PSMA-617 Cyclisation Products
1	Phenomenex^®^ Luna 150 × ⌀3 mm, 5 μm, 100 Å	0–5–15–20 min = 17–25–25–17%B 0.75 mL/min, B—0.1% TFA in acetonitrile	1.11 ± 0.05	7.26 ± 0.04	1.4–7.0	7.6–9.3
2	Phenomenex^®^ Jupiter 250 × ⌀4.6 mm, 5 μm, 300 Å	0–1–25–27–29–32 min = 5–5–50–95–95–5%B, 1 mL/min, B—0.1% TFA in acetonitrile [37]	3.64 ± 0.08	16.90 ± 0.10	4.0–16.7	17.2–18.8
3	Phenomenex^®^ Jupiter 250 × ⌀4.6 mm, 5 μm, 300 Å	0–20–25–25.01–30 min = 15–100–100–15–15% B, 1 mL/min, B—methanol [17]	3.54 ± 0.08	13.61 ± 0.72	3.7–13.2	14.2–15.1

## Data Availability

The data presented in this study are available upon request from the corresponding author. The data are not publicly available in accordance with the current rules of the Federal Medical Biological Agency of Russia for research conducted under a state assignment.

## Supplementary Materials

The following supporting information can be downloaded at: https://www.mdpi.com/article/10.3390/molecules28041884/s1, Figure S1: Typical radio-TLC chromatograms of [^177^Lu]Lu-PSMA-617 preparation (sample volume—1 mL, ^177^Lu activity in the sample—150 MBq, 10 μg of PSMA-617, pH 4.5, synthesis at 95 °C for 30 min) obtained with different methods; Figure S2: Typical radio-HPLC chromatograms of [^177^Lu]Lu-PSMA-617 preparation obtained at the EOS time with different HPLC methods; Figure S3: Typical radio-HPLC chromatograms of [^177^Lu]Lu-PSMA-617 preparation obtained 24 h after EOS time (Figure 3) with different HPLC methods; Figure S4: The radio-TLC chromatograms of [^177^Lu]Lu-PSMA-617 preparation over time (sample volume—1 mL, ^177^Lu activity in the sample—4800 MBq, 100 μg of PSMA-617, pH 4.5, synthesis at 95 °C for 30 min) obtained with different methods; Figure S5: Radio-HPLC chromatograms of [^177^Lu]Lu-PSMA-617 preparation obtained with HPLC method 1: A. [^177^Lu]Lu-PSMA-617 sample (^177^Lu activity in the sample—450 MBq, 100 μg of PSMA-617, pH 4.5, C(Na-acetate) = 0.03 mol/L, synthesis at 95 °C for 30 min) with addition of [^177^Lu]Lu^3+^ (pH 4.5, C(Na-acetate) = 0.03 mol/L) after EOS; B. sample “A” after CM cartridge; C. [^177^Lu]Lu-PSMA-617 sample (^177^Lu activity in the sample—450 MBq, 100 μg of PSMA-617, pH 4.5, C(Na-acetate) = 0.03 mol/L, synthesis at 95 °C for 30 min) after 6 h of storage at RT; D. sample “C” after CM cartridge; Figure S6: Time dependence of the decrease in [^177^Lu]Lu-PSMA-617 radiochemical purity value (sample volume—1 mL, ^177^Lu activity—5 GBq, 100 μg of PSMA-617, pH 4.5, C(Na-acetate) = 1 mol/L); Figure S7: Proposed pathway for the formation of the major mixed disulfide of DMSA with L-cysteine; Figure S8: The influence of addition of cysteine and DMSA in different molar ratios on the maintenance of [^177^Lu]Lu-PSMA-617 radiochemical purity over time. Samples volume—1 mL, ^177^Lu activity in the sample—440 MBq, 10 μg of PSMA-617, pH 4.5, C(Na-acetate) = 0.03 mol/L (RCP value is presented as mean ± SD, *n* = 3); Table S1: Published data on specific bimolecular rate constants (k, L·mol^−1^·s^−1^) for the reactions of different radicals with selected compounds in aqueous solution. Refs. [48,71,84,85,86] are cited in the Supplementary Materials.

## Sample Availability

Samples of the compounds are not available from the authors.
