# Supplementary material for: Radiolysis-Associated Decrease in Radiochemical Purity of 177Lu-Radiopharmaceuticals and Comparison of the Effectiveness of Selected Quenchers against This Process
Source: Molecules. 2023 Feb 16;28(4):1884. doi: 10.3390/molecules28041884 (PMC9967390; doi:10.3390/molecules28041884)
Supplement: Supplementary file 1 [file molecules-28-01884-s001.zip › molecules-2210520-supplementary.pdf]

Supplementary Materials

Radiolysis-associated decrease in radiochemical purity of ^{177}Lu-radiopharmaceuticals and comparison of the effectiveness of selected quenchers against this process

Anton Larenkov, Iurii Mitrofanov, Ekaterina Pavlenko and Marat Rakhimov

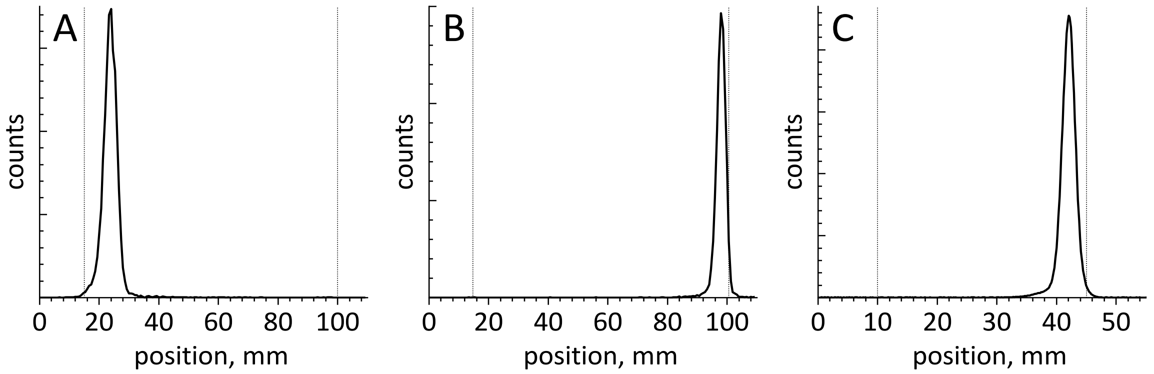

Figure S1. Typical radio-TLC chromatograms of [^{177}Lu]Lu-PSMA-617 preparation (sample volume – 1 mL, ^{177}Lu activity in the sample – 150 MBq, 10 μg of PSMA-617, pH 4.5, synthesis at 95°C for 30 minutes) obtained with different methods: A – method 1, iTLC-SG/0.05 M $H_3Citr_{aq.}$; B – method 2, iTLC-SG/ NH_3–Ethanol–H_2O (1:5:10); C – method 3, 5553 TLC Silica gel 60 / MeCN–H_2O (1:1).

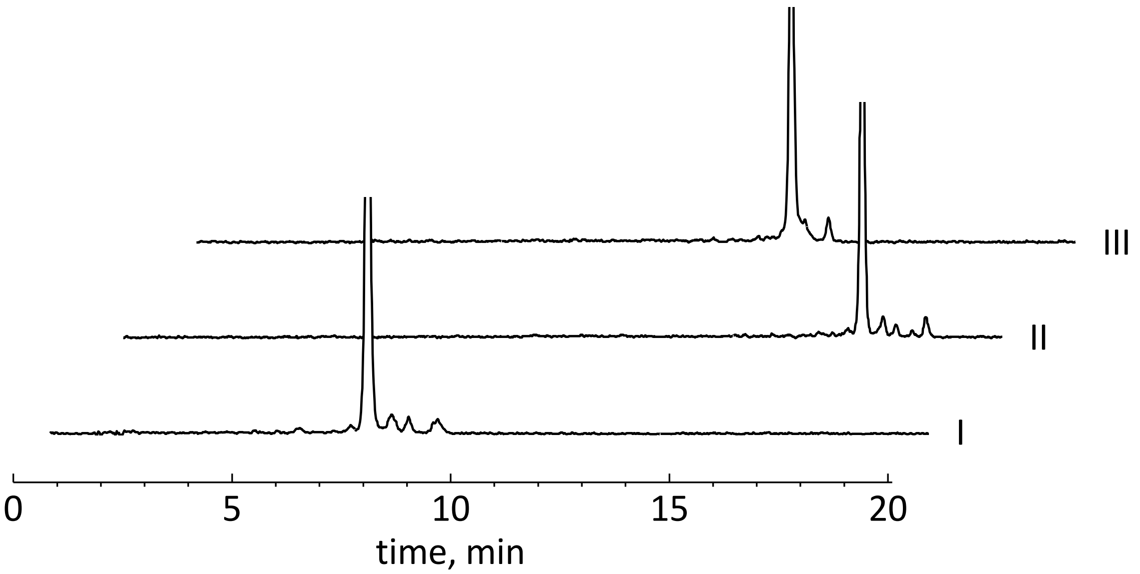

Figure S2. Typical radio-HPLC chromatograms of [^{177}Lu]Lu-PSMA-617 preparation obtained at the EOS time with different HPLC methods: I – method 1, II – method 2, III – method 3 (see *Materials and Methods*): sample volume – 1 mL, ^{177}Lu activity in the sample – 450 MBq, 100 μg of PSMA-617, pH 4.5, C(Na-acetate) = 0.03 mol/L, synthesis at 95°C for 30 minutes.

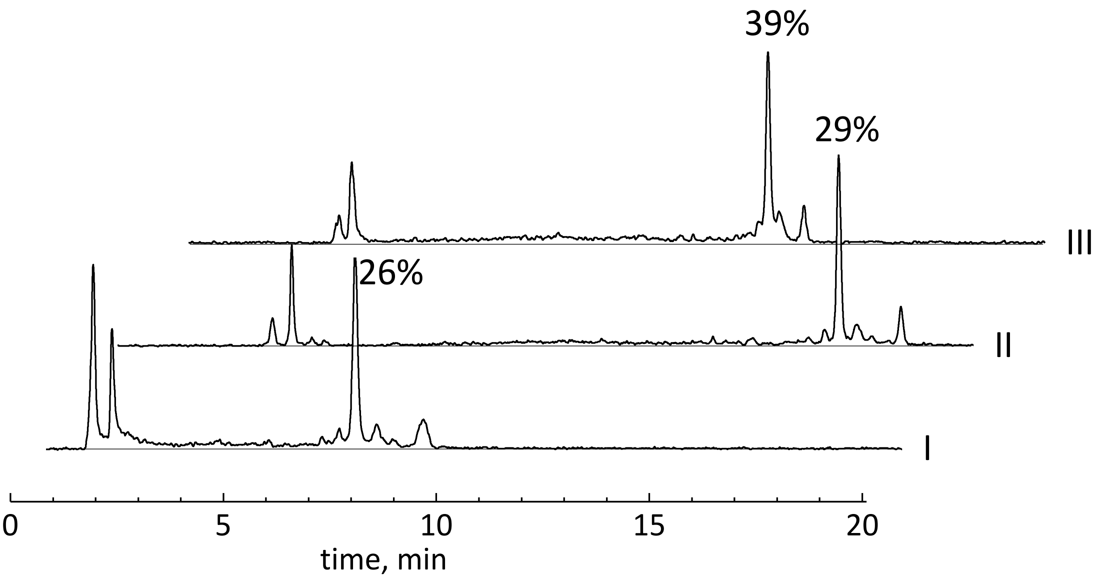

Figure S3. Typical radio-HPLC chromatograms of [^{177}Lu]Lu-PSMA-617 preparation obtained 24 h after EOS time (**Figure S2**) with different HPLC methods: I – method 1, II – method 2, III – method 3 (see *Materials and Methods*).

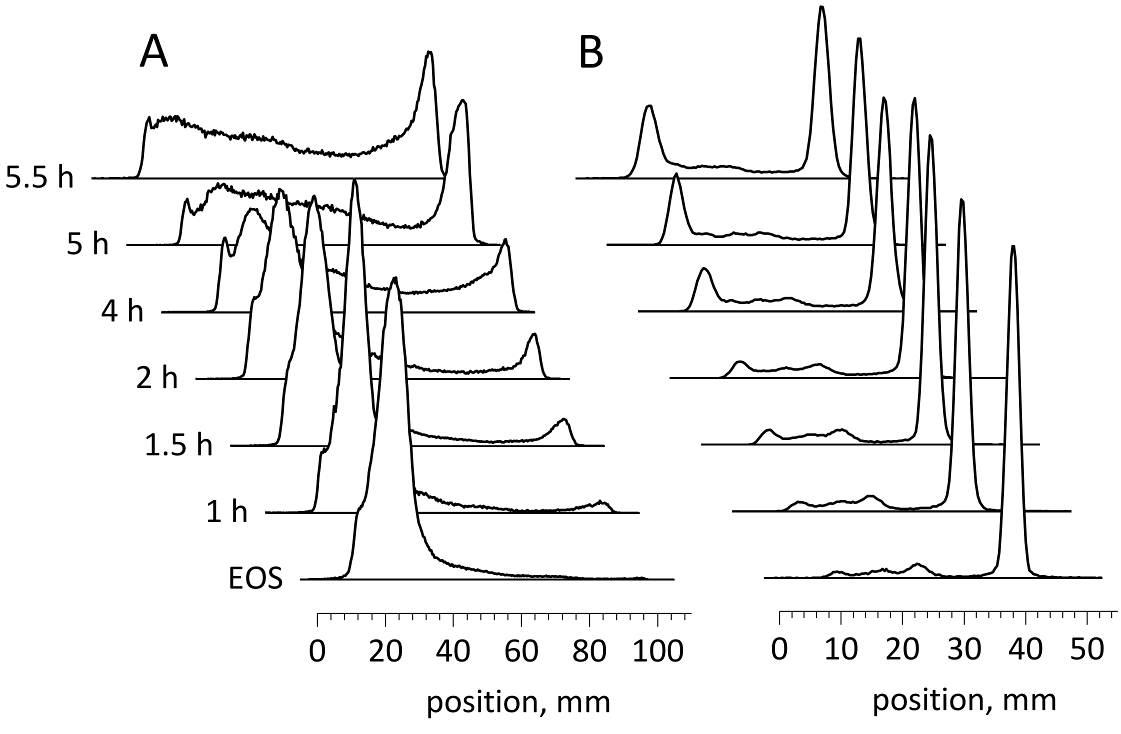

Figure S4. The radio-TLC chromatograms of [^{177}Lu]Lu-PSMA-617 preparation over time (sample volume – 1 mL, ^{177}Lu activity in the sample – 4800 MBq, 100 μg of PSMA-617, pH 4.5, synthesis at 95°C for 30 minutes) obtained with different methods: A – method 1, iTLC-SG/0.05 M $H_3Citr_{aq.}$; B – method 3, 5553 TLC Silica gel 60 / MeCN–H_2O (1:1).

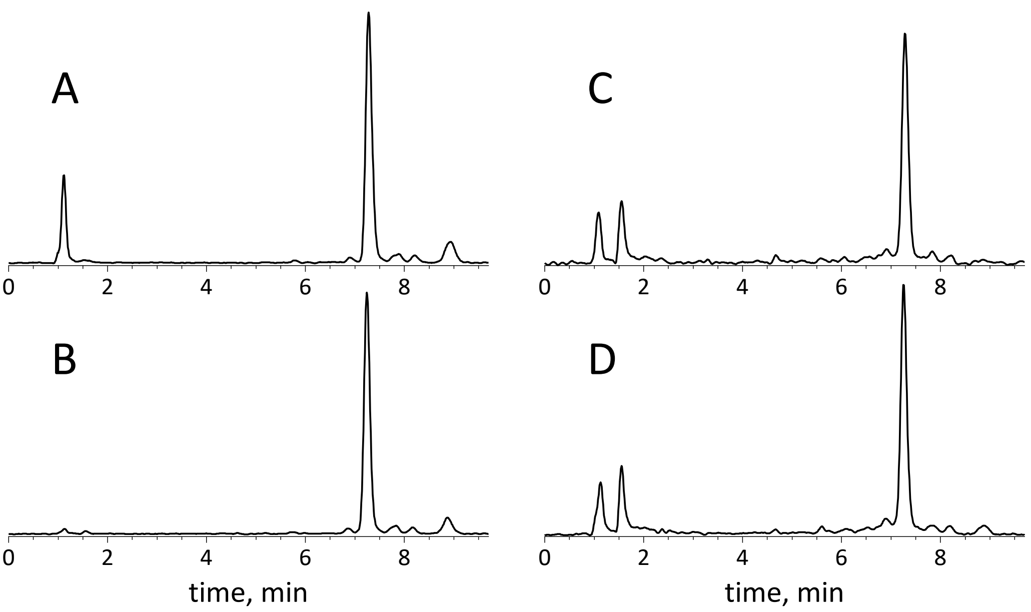

Figure S5. Radio-HPLC chromatograms of [^{177}Lu]Lu-PSMA-617 preparation obtained with HPLC method 1: **A.** [^{177}Lu]Lu-PSMA-617 sample (^{177}Lu activity in the sample – 450 MBq, 100 µg of PSMA-617, pH 4.5, C(Na-acetate) = 0.03 mol/L, synthesis at 95°C for 30 min) with addition of [^{177}Lu]Lu^{3+} (pH 4.5, C(Na-acetate) = 0.03 mol/L) after EOS; **B.** sample "A" after CM cartridge; **C.** [^{177}Lu]Lu-PSMA-617 sample (^{177}Lu activity in the sample – 450 MBq, 100 µg of PSMA-617, pH 4.5, C(Na-acetate) = 0.03 mol/L, synthesis at 95°C for 30 min) after 6 h of storage at RT; **D.** sample "C" after CM cartridge.

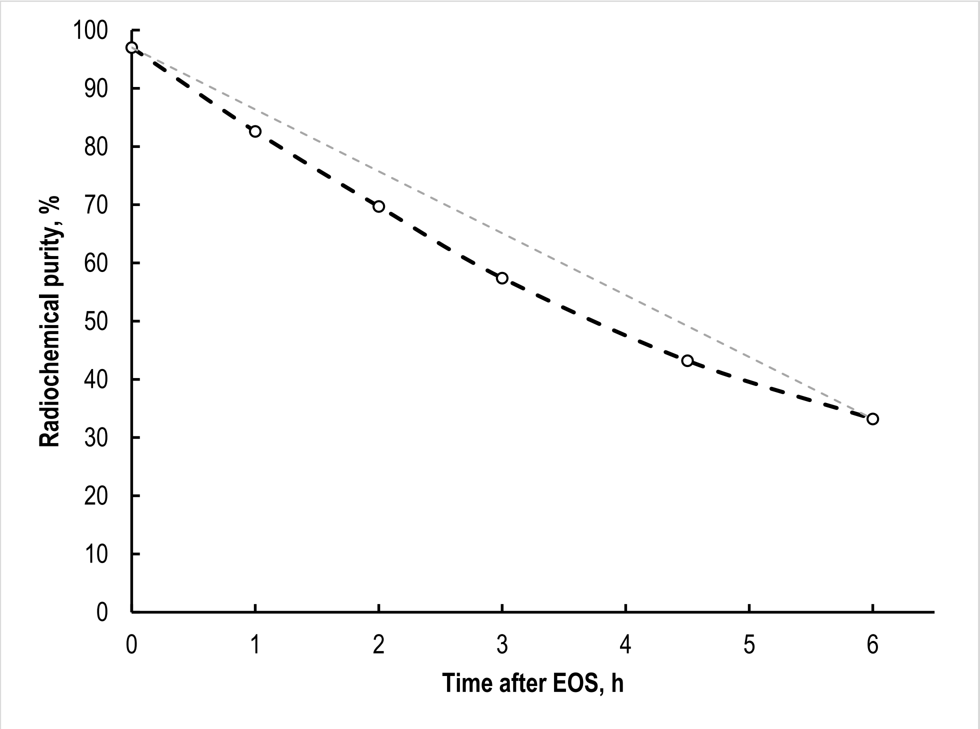

Figure S6. Time dependence of the decrease in [^{177}Lu]Lu-PSMA-617 radiochemical purity value (sample volume – 1 mL, ^{177}Lu activity – 5 GBq, 100 µg of PSMA-617, pH 4.5, C(Na-acetate) = 1 mol/L).

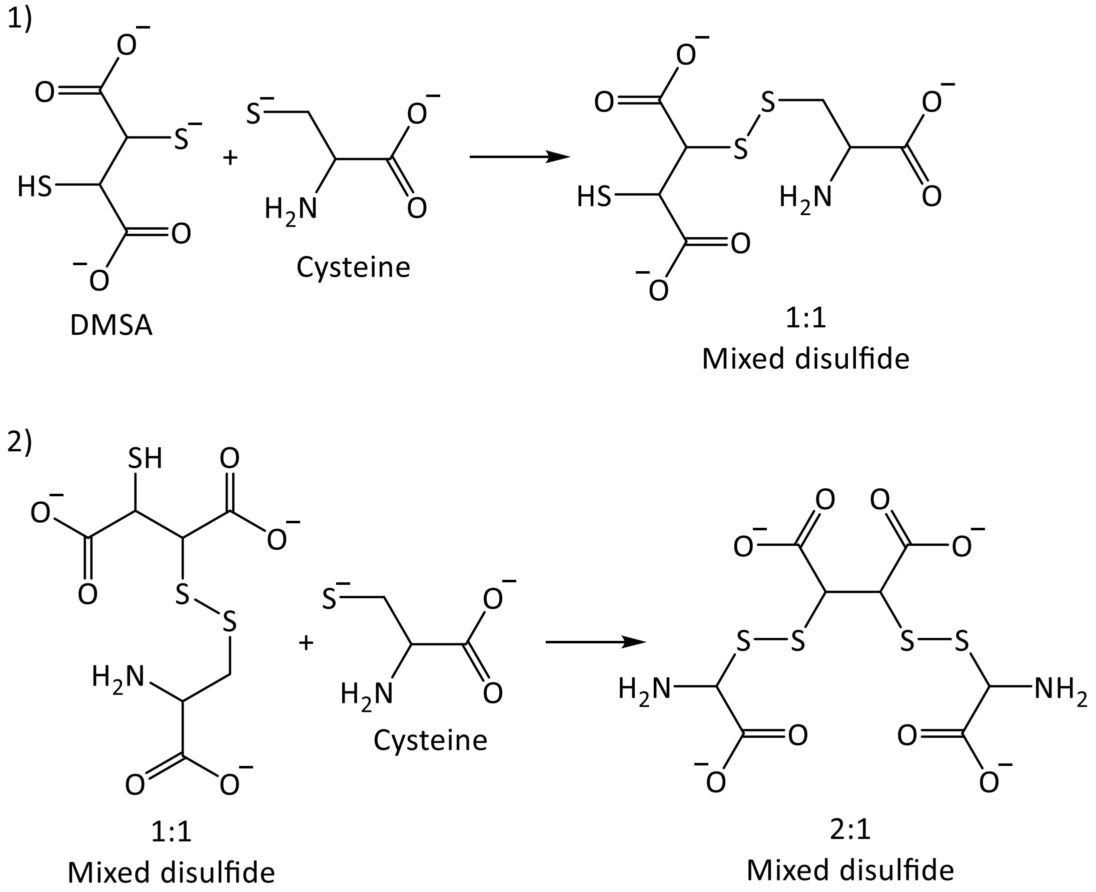

Figure S7. Proposed pathway for the formation of the major mixed disulfide of DMSA with L-cysteine.

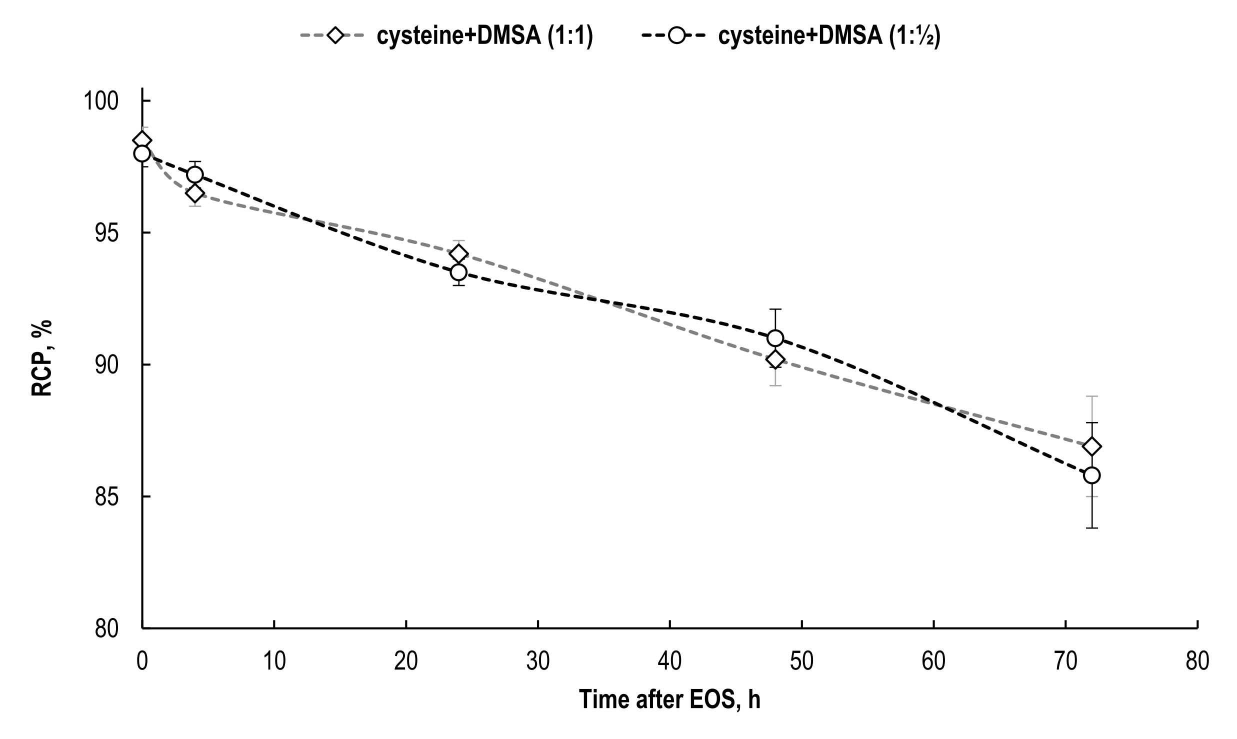

Figure S8. The influence of addition of cysteine and DMSA in different molar ratios on the maintenance of [^{177}Lu]Lu-PSMA-617 radiochemical purity over time. Samples volume – 1 mL, ^{177}Lu activity in the sample – 440 MBq, 10 µg of PSMA-617, pH 4.5, C(Na-acetate) = 0.03 mol/L (RCP value is presented as mean ± SD, n=3).

Table S1. Published data on specific bimolecular rate constants (k, L·mol^{-1}·s^{-1}) for the reactions of different radicals with selected compounds in aqueous solution.

**Compound**	**Radical**				Ref.
	**H·**	**·OH**	**·O^-**	**$e^-_{aq.}$**	
Ascorbic acid	1.0–1.6×10^{8}(pH 1)	–	–	–	[1]
	1.3×10^{8} (pH 1)	–	–	–	[2]
	–	7.2×10^{9} (pH 1)	–	–	[3]
Ascorbate ion	3×10^{8} (pH 7)	4.1×10^{9} (pH 11)<br>1.1–1.3×10^{10} (pH 7)<br>6.5×10^{9} (pH 7–8)	–	3.0–4.0×10^{8} (pH 7)	[1]
Gentisic acid	–	1.1×10^{10} (pH 7)	–	–	[4]
Ethanol	1.6×10^{7} (pH 7)<br>1.5–3.8×10^{7} (pH 1)	1.2×10^{9} (pH 6 –10.5)<br>7.2×10^{8} (pH 7)<br>1.1×10^{9} (pH 3,9,7)	–	<10^{5}	[3]
	1.7×10^{7} (pH 1)	1.9×10^{9} (pH 6 – 7)	1.2×10^{9} (pH 11 –14)	–	[1]
Acetic acid	1-2.5×10^{5} (pH 1)	1.4×10^{7} (pH 1)	–	1.8×10^{8} (pH 5.4)	[3]
Acetate ion	2.7×10^{5} (pH 7)	4.2×10^{7} (pH 10.7)<br>4.3-5.3×10^{7} (pH 9)	–	<10^{6} (pH 10)	[3]
	3.9×10^{5} (pH 7)	7.4×10^{7} (pH 10.7)	5×10^{7} (pH 14)	1.1×10^{6} (pH 9–11.5)	[1]
Nicotinamide	–	1.4×10^{9} (pH 5.9)<br>1.5×10^{9} (pH 9.0)	–	2.4×10^{10} (pH 7.5)	[1]
Cysteine	1.0×10^{9} (pH 6) – cystine formation<br>1.2×10^{8} (pH 6) – H_2S formation	7.9×10^{9} (pH 1)	–	8.7×10^{9} (pH 6.3)<br>7.5×10^{7} (pH 11.6)	[3]
	1.2×10^{9} (pH 1)	1.9 × 10^{10} (pH 5.8)<br>3.5–4.7 × 10^{10} (pH 7)	–	1.0×10^{10} (pH 7)<br>1.3×10^{10} (pH 5.8)	[1]
Methionine	3.5×10^{8} (pH 1)	8.5×10^{9} (pH 6–7)<br>8.1×10^{9} (pH 5.5–5.7)	–	4.5×10^{7} (pH 7.3)<br>3.5×10^{7} (pH 6)	[1]
	-	3.7×10^{9} (pH 2)<br>5.1×10^{9} (pH 7)	–	3.5×10^{7} (pH 6)	[3]
Adenine	6.0×10^{8} (pH 7)	5.3×10^{8} (pH 2)<br>2.3×10^{9} (pH 5)<br>3.0×10^{9} (pH 7.4)	–	6.2×10^{9} (pH 7.7)	[3]
	1.0×10^{8} (pH 7)	8.8×10^{8} (pH 2–2.2)	–	9.0×10^{9} (pH 7)	[1]
Vanillin	–	3.3×10^{9} (pH 9)	–	–	[5]
Thymine	5.0×10^{8} (pH 1)<br>6.8×10^{8} (pH 0.65)	6.4×10^{9} (pH 6–9)	4×10^{8} (pH 13)	1.8×10^{10} (pH 5.5–6.0)	[1]
	–	3.1×10^{9} (pH 1-7)<br>2.2×10^{9} (pH 0.7–7)	–	1.8×10^{10} (pH 5.5)<br>1.7×10^{10} (pH 6)	[3]
Uracil	3.0×10^{9} (pH 7)	3.1×10^{9} (pH 5–7)<br>4.1×10^{9} (pH 9)	–	7.7×10^{9} (pH 6.4)	[3]
	4.7×10^{8} (pH 2.5)<br>2.8×10^{8} (pH 2)	4.7×10^{8} (pH 2.5)<br>2.8×10^{8} (pH 2)	–	1.5×10^{10} (pH 7)	[1]

1. Buxton, G. V.; Greenstock, C.L.; Helman, W.P.; Ross, A.B. Critical Review of Rate Constants for Reactions of Hydrated Electrons, Hydrogen Atoms and Hydroxyl Radicals (·OH/·O− in Aqueous Solution. *J. Phys. Chem. Ref. Data* **1988**, *17*, 513–886, doi:10.1063/1.555805.
2. Madden, K.P.; Mezyk, S.P. Critical Review of Aqueous Solution Reaction Rate Constants for Hydrogen Atoms. *J. Phys. Chem. Ref. Data* **2011**, *40*, doi:10.1063/1.3578343.
3. Anbar, M.; Neta, P. A Compilation of Specific Bimolecular Rate Constants for the Reactions of Hydrated Electrons, Hydrogen Atoms and Hydroxyl Radicals with Inorganic and Organic Compounds in Aqueous Solution. *Int. J. Appl. Radiat. Isot.* **1967**, *18*, 493–523, doi:10.1016/0020-708X(67)90115-9.
4. Joshi, R.; Gangabhagirathi, R.; Venu, S.; Adhikari, S.; Mukherjee, T. Antioxidant Activity and Free Radical Scavenging Reactions of Gentisic Acid: In-Vitro and Pulse Radiolysis Studies. *Free Radic. Res.* **2012**, *46*, 11–20, doi:10.3109/10715762.2011.633518.
5. Mahal, H.S.; Badheka, L.P.; Mukherjee, T. Radical Scavenging Properties of a Flavouring Agent-Vanillin. *Res. Chem. Intermed.* **2001**, *27*, 595–604, doi:10.1163/156856701317051699.
